# The relationship between isolated hypertension with brain volumes in UK Biobank

**DOI:** 10.1002/brb3.2525

**Published:** 2022-04-01

**Authors:** Danielle Newby, Laura Winchester, William Sproviero, Marco Fernandes, Upamanyu Ghose, Donald Lyall, Lenore J. Launer, Alejo J. Nevado‐Holgado

**Affiliations:** ^1^ Department of Psychiatry Warneford Hospital, University of Oxford Oxford UK; ^2^ Institute of Health and Wellbeing University of Glasgow Scotland UK; ^3^ National Institute on Aging Bethesda Maryland USA; ^4^ Big Data Institute University of Oxford Oxford UK

**Keywords:** brain health, hypertension, isolated hypertension

## Abstract

**Background:**

Hypertension is a well‐established risk factor for cognitive impairment, brain atrophy, and dementia. However, the relationship of other types of hypertensions, such as isolated hypertension on brain health and its comparison to systolic‐diastolic hypertension (where systolic and diastolic measures are high), is still relatively unknown. Due to its increased prevalence, it is important to investigate the impact of isolated hypertension to help understand its potential impact on cognitive decline and future dementia risk. In this study, we compared a variety of global brain measures between participants with isolated hypertension to those with normal blood pressure (BP) or systolic‐diastolic hypertension using the largest cohort of healthy individuals.

**Methods:**

Using the UK Biobank cohort, we carried out a cross‐sectional study using 29,775 participants (mean age 63 years, 53% female) with BP measurements and brain magnetic resonance imaging (MRI) data. We used linear regression models adjusted for multiple confounders to compare a variety of global, subcortical, and white matter brain measures. We compared participants with either isolated systolic or diastolic hypertension with normotensives and then with participants with systolic‐diastolic hypertension.

**Results:**

The results showed that participants with isolated systolic or diastolic hypertension taking BP medications had smaller gray matter but larger white matter microstructures and macrostructures compared to normotensives. Isolated systolic hypertensives had larger total gray matter and smaller white matter traits when comparing these regions with participants with systolic‐diastolic hypertension.

**Conclusions:**

These results provide support to investigate possible preventative strategies that target isolated hypertension as well as systolic‐diastolic hypertension to maintain brain health and/or reduce dementia risk earlier in life particularly in white matter regions.

## Graphical abstract

The relationship between brain health and different types of hypertensions such as isolated hypertension is unknown. Using UK Biobank, we showed that participants with isolated hypertension taking BP medications had smaller gray matter but larger white matter microstructures and macrostructures compared to normotensives. Isolated systolic hypertensives had larger total gray matter and smaller white matter traits when comparing these regions with participants with systolic‐diastolic hypertension. These results provide support to investigate possible preventative strategies that target isolated hypertension.

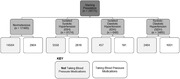

## 1 INTRODUCTION

Hypertension in midlife is a well‐established risk factor for cognitive impairment and dementia (Lennon et al., 2019; Sharp et al., 2011). As there is currently no cure for dementia, there is great interest in understanding the roles of modifiable risk factors such as hypertension in slowing or preventing the disease (Livingston et al., 2020). The current European guidelines for hypertension recommend that adults are diagnosed with suspected hypertension based on a threshold of systolic blood pressure (BP) ≥140 mm Hg and diastolic BP ≥90 mm Hg (Williams et al., 2018). This is known as combined systolic‐diastolic hypertension (SDH) and is associated with a predominant rise in arteriolar resistance and increased stiffness of the large arteries resulting in increases in systolic and diastolic BP. However, there are other types of hypertensions. Isolated systolic hypertension (ISH) occurs when there is only an increase in stiffness of the aorta and other large arteries but no rise in arteriolar resistance. It is defined as systolic BP greater than 140 mm Hg and diastolic BP is less than 90 mm Hg. In contrast, a predominant rise in arteriolar resistance but with normal or low arterial stiffness can lead to isolated diastolic hypertension (IDH) and is defined as diastolic BP ≥90 mm Hg and systolic BP < 140 mm Hg (Verdecchia & Angeli, 2005)

ISH is the most common form of hypertension in older populations due to hardening and reduced elasticity of arteries resulting in an increase in pulse pressure and a decrease in diastolic BP with advancing age (Franklin et al., 2001). On the other hand, IDH is a largely unrecognized subtype of hypertension, more common in the younger ages (Franklin et al., 2001; Sagie et al., 1993). All three different types of hypertensions are independently associated with increased risk of stroke, heart disease/failure, and many others diseases (Guichard et al., 2011; Os et al., 2006; Qureshi et al., 2002). All these disease are also associated with increased risk of dementia (Garfield et al., 2021; Li et al., 2020; Livingston et al., 2020; Sierra, 2020).

There are numerous population studies examining the relationship between BP and risk of dementia. Although there are inconsistences, the studies do suggest that high BP (in particular high systolic BP) maybe a risk factor for cognitive decline and dementia risk (Lane et al., 2019; McGrath et al., 2017; Walker et al., 2019). These studies are supported by randomized controlled trials such as SYST‐Eur trial which showed treatment of ISH in elderly persons decreased dementia risk (Forette et al., 1998). The SPRINT‐MIND trial also showed intensive treatment of systolic BP resulted in a smaller decrease of total brain volume and smaller increase of cerebral white matter lesions volumes (Nasrallah et al., 2019).

The mechanisms underlying the associations between hypertension and cognitive impairment or dementia remain unclear. There is increasing evidence to suggest brain imaging studies can offer further insight between the links between the heart and the brain as well as cognitive decline and future dementia risk. There are growing numbers of studies which examine the relationship between hypertension and BP and its impact on the brain using UK Biobank (Ferguson et al., 2020; Lyall et al., 2016; Newby et al., 2021). These studies and others highlight that people with hypertension (and also other vascular risk factors (Cox et al., 2019)) have smaller volumes of brain tissue, gray matter (Jennings et al., 2012) in specific regions such as the hippocampus (Korf et al., 2004) and increased white matter hyperintensities (Wartolowska & Webb, 2021).

Due to the potential implications of isolated hypertension on cognitive decline and dementia risk, it is important to define the impact of isolated hypertension on the brain in a large cohort of nondemented individuals. There is a lack of studies, which focus on isolated hypertension, and its impact compared with those with normal BP and those with SDH. We hypothesize that those with isolated hypertension will have poorer brain health measures compared to those with normal BP. Additionally, those with isolated hypertension will have better brain health measures compared with those with both high systolic and diastolic BP under the assumption that having both high systolic and diastolic is more detrimental. The objective of this study is to compare brain volumes between individuals with isolated (systolic and diastolic) hypertension in the UK Biobank cohort with normotensives and those with SDH. This analysis provides a novel investigation into the impact of isolated hypertension on the brain in the largest population of people with no clinical diagnosis of dementia or cognitive impairment.

## 2 METHODS

### 2.1 Study design

A cross‐sectional study of 29,775 participants with BP measurements and brain MRI data in UK Biobank was used to determine the association between isolated systolic and diastolic hypertension with several global brain volumes, gray subcortical, white matter micro, and macrostructures volumes associated with cognitive decline and dementia utilized in previous works (Lyall et al., 2019; Newby et al., 2021).

### 2.2 Setting

UK Biobank is a large prospective cohort of over half a million participants. All participants, aged between 37 and 73, initially attended baseline assessment visit from 2006 to 2010 where they completed a series of physical, sociodemographic, cognitive, and medical assessments (Sudlow et al., 2015). Subsets of participants have also been followed up with 100,000 participants being followed up from 2014 to 2023. Participants in this follow‐up have or will have the typical assessments as with baseline visit but will undergo whole body imaging including magnetic resonance imaging (MRI) brain imaging. UK Biobank received ethical approval from the Research Ethics Committee (11/NW/0382). Volunteers gave informed consent for their participation.

### 2.3 Participants

Participants who attended the assessment center for an MRI brain scan with valid systolic and diastolic BP measurements were included in this study. These participants also provided demographic, health, and socioeconomic information using touchscreen questionnaires as well as taking part in a nurse‐led interview asking questions about medical history and medications. Two BP measurements were performed on each participant using automated Omron Digital BP monitor. For this work, only the second BP measurement was used as there is evidence the first reading can overestimate BP due to white coat syndrome (Einstadter et al., 2018). Based on our previous work (Newby et al., 2021), participants who reported they had any neurodegenerative or related diseases were excluded from this analysis (*n* = 968). A full list of these diseases and UK Biobank field codes for all variables used in this study can be found in Tables S1 and S2. We removed participants with body mass index (BMI) <18.5 kg/m^2^ (*n* = 506) and those with no valid BP measurements resulting in 29,775 participants.

**TABLE 1:** Characteristics of UK Biobank participants at imaging visit included stratified by hypertensive state

	Normotensive (*n* = 17,488)	Isolated diastolic hypertensive (*n* = 648)	Isolated systolic hypertensive (*n* = 8174)	Systolic‐diastolic hypertensive (*n* = 3465)	*n*
Demographics					
Age years (mean [SD])	62.1 (7.50)	60.1 (7.35)	66.6 (6.68)	63.5 (7.29)	29,775
Gender (male *N* [%])	7293 (41.7%)	356 (54.9%)	4389 (53.7%)	2089 (60.3%)	29,775
Ethnicity (white *N* [%])	16,884 (96.8%)	609 (94.3%)	7967 (97.7%)	3350 (97.0%)	29,694
Education: Degree *N* (%)	8905 (51.3%)	315 (48.9%)	3626 (44.9%)	1584 (46.3%)	29,508
Townsend Deprivation Index Decile (mean [SD])	5.55 (2.87)	5.65 (2.92)	5.31 (2.83)	5.41 (2.86)	29,750
Assessment center *N* (%)					29,775
Cheadle	12,155 (69.5%)	480 (74.1%)	5128 (62.7%)	2336 (67.4%)	20,099
Reading	2426 (13.9%)	65 (10.0%)	868 (10.6%)	249 (7.19%)	3608
Newcastle	2907 (16.6%)	103 (15.9%)	2178 (26.6%)	880 (25.4%)	6068
Body mass index kg/m^2^ (mean [SD])	26.0 (4.18)	28.5 (5.05)	27.0 (4.30)	28.0 (4.48)	29,775
Smoking status (ever/current: *N* [%])	6347 (36.5%)	218 (34.0%)	3178 (39.2%)	1271 (37.1%)	29,535
Diastolic blood pressure mm Hg (mean [SD])	73.8 (7.74)	92.9 (3.30)	80.0 (6.51)	95.8 (5.32)	29,775
Systolic blood pressure mm Hg (mean [SD])	124 (10.3)	133 (5.34)	152 (10.6)	159 (14.3)	29,775
Taking blood pressure medications (*N* [%])	2904 (16.6%)	191 (29.5%)	2616 (32.0%)	1001 (28.9%)	29,775
Hypercholesterolemia (*N* [%])	3575 (20.4%)	136 (21.0%)	2615 (32.0%)	867 (25.0%)	29,775
Diabetes (*N* [%])	814 (4.65%)	30 (4.63%)	600 (7.34%)	180 (5.19%)	29,775
Brain volumes					
Total brain volume mm^3^ (mean [SD))	1,164,177 (110,236)	1,184,290 (111,312)	1,155,079 (111,485)	1,174,346 (112,863)	29,768
Gray matter mm^3^ (mean [SD))	618,366 (55,063)	627,113 (55,708)	608,884 (55,570)	619,085 (56,123)	29,771
WMH mm^3^ (mean [SD])	3722 (4184)	4099 (4583)	5529 (5368)	5233 (5323)	28,357
gFA units *M* (SD)	0.06 (0.53)	0.05 (0.55)	−0.08 (0.57)	−0.06 (0.58)	28,025
gMD units *M* (SD)	−0.06 (0.42)	−0.08 (0.45)	0.09 (0.48)	0.06 (0.48)	28,025
Ventricular CSF mm^3^ (mean [SD])	34,179 (15,267)	33,476 (14,809)	39,003 (16,500)	37,753 (16,410)	29,636
Hippocampus mm^3^ (mean [SD])	3862 (427)	3906 (426)	3804 (441)	3864 (452)	29,739
Accumbens mm^3^ (mean [SD])	451 (104)	464 (106)	426 (104)	442 (105)	29,760
Amygdala mm^3^ (mean [SD])	1248 (215)	1255 (219)	1248 (218)	1261 (219)	29,755
Pallidum mm^3^ (mean [SD])	1780 (216)	1791 (220)	1769 (230)	1794 (232)	29,706
Putamen mm^3^ (mean [SD])	4814 (560)	4927 (564)	4754 (574)	4853 (593)	29,733
Caudate mm^3^ (mean [SD])	3472 (413)	3516 (410)	3458 (424)	3511 (434)	29,730
Thalamus mm^3^ (mean [SD])	7697 (722)	7813 (719)	7582 (726)	7707 (736)	29,711

*Note*: Normotensive: diastolic blood pressure BP < 90 mm Hg and systolic BP < 140 mm Hg; Isolated diastolic hypertensive (IDH): diastolic blood pressure BP ≥90 mm Hg and systolic BP < 140 mm Hg; Isolated systolic hypertensive (ISH): systolic BP ≥140 mm Hg and diastolic BP < 90 mm Hg; systolic‐diastolic hypertension (SDH): diastolic blood pressure BP ≥90 mm Hg and systolic BP ≥140 mm Hg.

### 2.4 Variables

#### 2.4.1 Isolated systolic and diastolic hypertension

**FIGURE 1:**
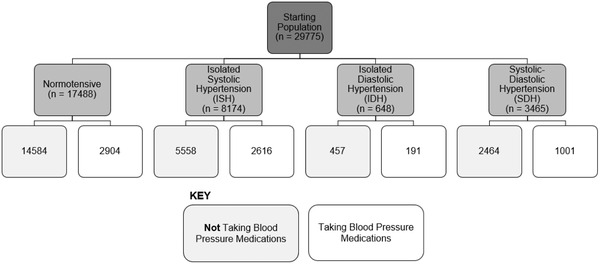
Numbers of participants in different hypertensive sub classes and split by blood pressure medication usage used in this study

SDH was defined as participants with a diastolic BP ≥90 mm Hg and systolic BP ≥140 mm Hg (*n* = 3465). Participants were defined as having ISH if they had systolic blood pressure (SBP) ≥140 mm Hg but a diastolic blood pressure (DBP) <90 mm Hg (*n* = 8174). Participants were defined as having IDH if they had a DBP ≥90 mm Hg but a SBP <140 mm Hg (*n* = 648). These groups were further split into two groups depending on whether they reported they were taking BP medications or not (Figure 1).

#### 2.4.2 Brain volumes

Brain MRIs were acquired on a Siemens Skyra 3 T scanner with a standard Siemens 32‐channel head coil. We utilized imaging derived phenotypes (IDPs) derived from the raw brain MRI images which were generated using an image‐processing pipeline developed and quality controlled centrally by UK Biobank (Alfaro‐Almagro et al., 2018). To reduce risk of type‐1 error through multiple testing, we selected a priori phenotypes known to underlie some degree of cognitive impairment throughout the lifespan as with previous works (Lyall et al., 2019). In this study, we included total brain volume, gray matter volume, and white matter hyperintensity (WMH) volume (potentially indicative of poorer cerebrovascular health) (Wardlaw et al., 2015). Additionally, we analyzed subcortical volumes (accumbens, amygdala, caudate, hippocampus, pallidum, putamen, and thalamus—left and right hemispheres due to potential side specificity differences (Tank et al., 2021) and average measures), and latent measures of tract‐averaged fractional anisotropy (FA) and mean diffusivity (MD) of all the white matter tracts. FA and MD are two metrics imaged with diffusion‐tensor imaging (DTI) indicative of white matter tract microstructural integrity: higher FA values suggest better health, whereas higher MD suggests worse white matter tract health. Due to the high correlation of the white matter microstructural properties across the brain of individual regions of FA and MD, we created single general latent measures of FA and MD using confirmatory factor analysis. Following other published works (Cox et al., 2019, 2016), we created two latent measures of general white matter fractional anisotropy (gFA) and mean diffusivity (gMD). Outlier data points, defined as being further than ±4 SD from the mean, were excluded (<1% of values).

### 2.5 Covariates

For all analysis, models were adjusted for age at assessment visit, sex, education, deprivation (Townsend index), ethnicity, assessment center, BMI, smoking status, diabetes, and hyperlipidemia. We included sex × age and sex × age^2^ as covariates to correct for the interactions between sex, age, and age^2^ (nonlinear effects), and we additionally included specific MRI scanner variables for head size and head position using the *x*‐, *y*‐ and *z*‐axis position coordinates which were mean centered.

Age at assessment was used in whole years, and gender was self‐reported as male or female. Educational qualifications were self‐reported and were dichotomized into whether participants held a university/college degree or not. Self‐reported ethnicity was dichotomized into white or nonwhite and if was missing was obtained from the baseline assessment visit. Assessment center was a multilevel variable of the assessment centers utilized for the repeated imaging visits (*n* = 3). Townsend deprivation index calculated before the baseline visit and was split into deciles. BMI was constructed from valid height and weight measurements calculated by UK Biobank and was used as a continuous measure. Smoking status was self‐reported and dichotomized into never smoked or ever smoker (current or former). For diabetes and hyperlipidemia diagnosis, a combination of self‐reported and clinical records were used. Where participants responded ‘‘Do not know’’ or ‘‘Prefer not to answer’’ these were treated as missing (< 1%) and missing data were not imputed. Multicollinearity was assessed, and all variables had acceptable variance inflation factor (VIF) values below 10 with most variables with VIF below two.

### 2.6 Statistical methods

In this study, all analyses were performed using R version 4.0.2. Descriptive statistics were generated to characterize the study cohort with respect to all study variables (see Table 1). We used one‐way analysis of variance and *χ* ^2^tests to compare normotensive, hypertensive, and isolated hypertensive participants on continuous and categorical variables. Linear regression models were used to estimate the association between hypertensive groups with brain volumes while adjusting for other covariates. All outcome variables were normally distributed apart from WMH, which was log transformed to make it normally distributed prior to analysis. Standardized beta coefficients are reported for all analyses to facilitate comparison of associations across the brain volumes. All *p*‐values were adjusted for multiple testing using false discovery rate (FDR).

A variety of statistical comparisons were carried out to define the relationship between isolated hypertension and brain volumes. In the first analysis, we compared normotensive participants with participants either with ISH or IDH stratified by medication use. We stratified by medication use to determine if people with either ISH or IDH had different brain volumes compared to people with normal BP and no history of hypertension. By not stratifying by BP medication use, this would mean those people taking BP medications but with normal BP would be classed as “normotensive” when in fact, they are not, so these participants should not be included in the normotensive reference group. Before the analysis of IDH, participants with high systolic BP were excluded from the analysis and those with high diastolic BP were excluded before the analysis of ISH. In the second analysis, we compared participants with isolated hypertension (either systolic or diastolic) with participants with SDH (i.e., both high systolic and diastolic BP) stratified by medication use. By having the reference group set as those with isolated hypertension, we can compare brain volumes between isolated and SDH participants.

## 3 RESULTS

In this study, 29,775 UK Biobank participants who had an MRI brain scan, valid BP and BMI measurements, and no prevalent neurological disorders were included. Participants were aged between 44 and 82 (*M* = 63.44, SD = 7.53) years containing 53% females. The characteristics stratified by hypertensive state for the participants used in this study are presented in Table 1. In Table 1, participants with isolated diastolic hypertension are younger, more likely to be male and nonwhite, be overweight, less likely to smoke, and less likely to be diabetic or have high cholesterol compared to all other groups. Participants with ISH are on average older and have a lower BMI with a lower proportion of males compared to those with IDH or SDH. Compared to all other groups, participants with ISH are more likely to be diabetic, have high cholesterol, less likely to have a degree, be taking BP medications, and more likely to smoke. As expected, the isolated systolic and diastolic hypertensive groups have higher systolic and diastolic BP, respectively. Further stratification by BP medication showed further differences between the different groups in this population (Table S3).

There were differences between brain measures across all groups; however, it must be noted that Table 1 shows raw values for brain measures which may be confounded by age‐sex differences. The level of missingness for the variables in Table 1 was <1% for all the demographics, whereas the highest missingness for the brain volumes was 5%–6% for WMH, gFA, and gMD.

### 3.1 Differences in brain volumes between normotensive and isolated systolic hypertensive participants

**FIGURE 2:**
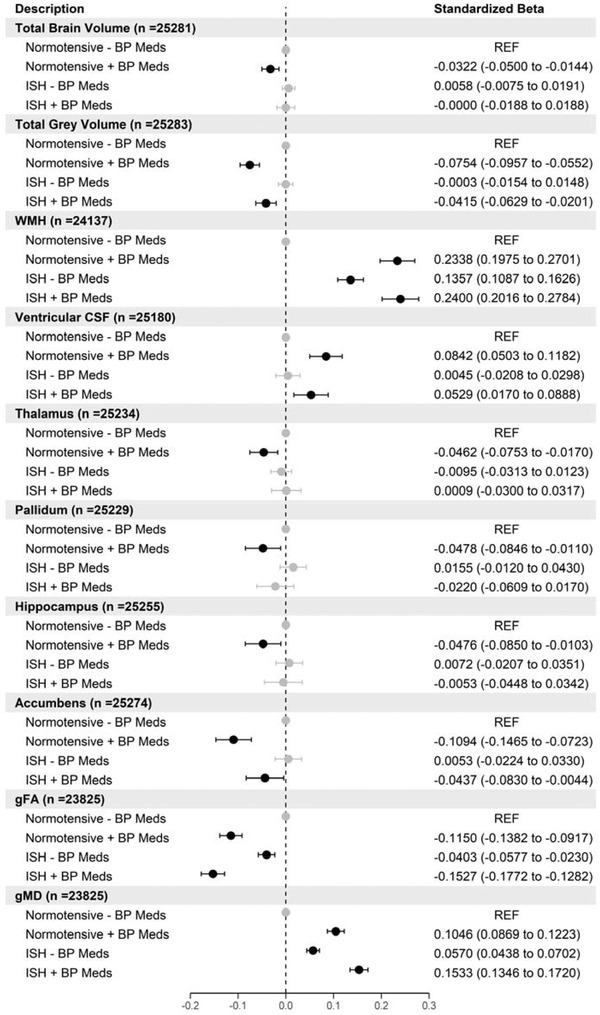
Forest plot showing the association of different brain volumes with isolated systolic hypertension (ISH) versus normotensive participants stratified by blood pressure medication use. Points in black are statistically significant (FDR *p* < .05) standardized betas (*n* = 25,662)

In this analysis, we compared brain volumes between normotensive participants not taking BP medications (*n* = 14,584) with participants with either (1) low BP but taking BP medications (*n* = 2904), (2) participants with ISH taking BP medications (*n* = 2616), and (3) participants with ISH not taking BP medications (*n* = 5558). Those with high diastolic BP (>90 mm Hg) were removed from the analysis to eliminate the effects of these participants in the comparisons (*n* = 4113). Compared with normotensives, those with ISH and taking BP medications have smaller total gray matter volumes (standardized *β* = −.04 SDs [95% CI −0.06 to −0.02]), larger ventricular CSF (*β* = .05 [95% CI 0.02–0.09]), and larger WMH (*β* = .24 [95% CI 0.20–0.28]), but there was no difference in total brain volumes. These participants also had smaller accumbens, lower gFA but higher gMD values compared to normotensives (Figure 2). Compared to the normotensive group, those with ISH not taking medications only differed in WMH, gFA, and gMD where participants with ISH taking medications had significantly larger WMH (*β* = .14 [95% CI 0.11–0.16]), larger gMD (*β* = .06 [95% CI 0.04–0.07]), and smaller gFA values (*β* = −.04 [95% CI −0.06 to −0.02]). Interestingly, there were significant associations among normotensive participants taking BP medications across all global brain measures, white matter structures, and the majority of subcortical regions. There were no differences between any of the groups compared with normotensives not taking BP medications for the caudate, amygdala, and putamen subcortical regions (Table S4).

As there is evidence in the literature to suggest side specificity of subcortical brain hemispheres (Tank et al., 2021), we repeated the above analysis for each subcortical region for the left and right sides (Figures S1 and S2). This analysis showed that for participants with isolated hypertension, there were no side specific differences for the accumbens. For normotensive individuals taking BP medications, the thalamus and the hippocampus did not show differences in hemispheres. The pallidum, amygdala, accumbens, and hippocampus regions showed differences between right and left hemispheres for participants with normal BP but taking BP medications. We also analyzed the white matter tract‐specific microstructure regions used to create then latent factors of gFA and gMD (Figures S3 and S4). The majority of the microstructure regions replicated the results of the latent factors of gFA and gMD in Figure 2.

### 3.2 Differences in brain volumes between normotensive and isolated diastolic hypertensive participants

Next, we investigated the relationship between IDH with brain measures. We compared brain volumes between the reference group of normotensive participants (*n* = 14,584) with participants with low BP but taking BP medications (*n* = 2904), participants with IDH taking BP medications (*n* = 191), and participants with IDH not taking BP medications (*n* = 457). As with the previous analysis, participants with high systolic BP (>140 mm Hg) were removed from the analysis (*n* = 11,639).

**FIGURE 3:**
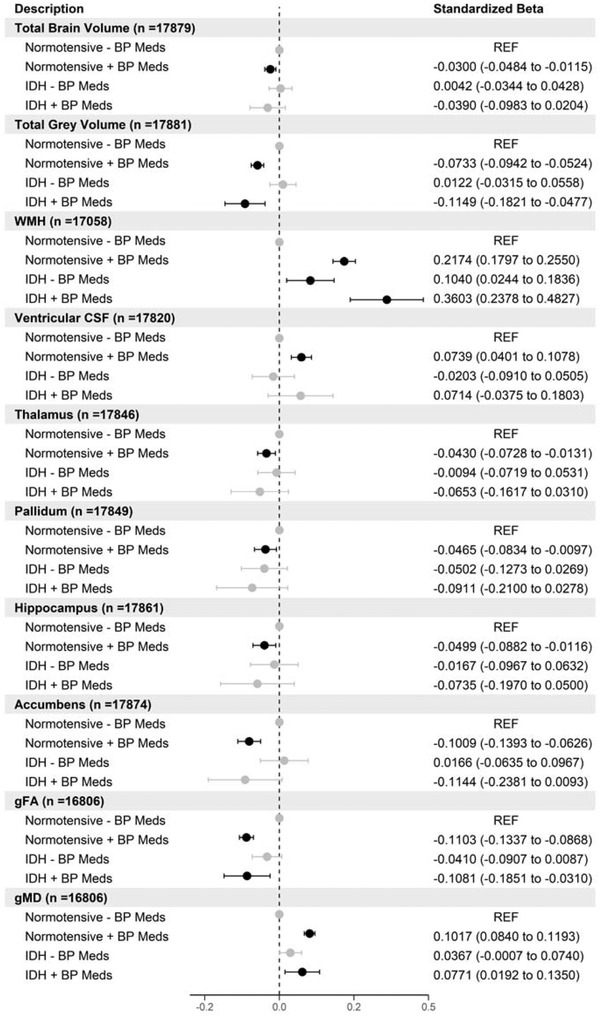
Forest plot showing the association of different brain volumes with isolated diastolic hypertension (IDH) versus normotensive participants stratified by blood pressure medication use. Points in black are statistically significant (FDR *p* < .05) standardized betas (*n* = 18,136)

As shown in Figure 3, those with IDH and taking BP medications have smaller total gray matter volumes (*β* = −.12 [95% CI −0.18 to −0.05]), smaller gFA values (*β* = −.11 [95% CI −0.19 to −0.03]), larger WMH (*β* = .36 [95% CI 0.24–0.48]), and larger gMD values (*β* = .08 [95% CI 0.02–0.14]). Participants who were normotensive but taking BP medications were associated with smaller global brain volumes, smaller subcortical volumes, smaller gFA values and larger WMH, ventricular CSF, and gMD values. Results for certain subcortical brain volumes (caudate and putamen and amydgala) showed no significant differences between any groups therefore were not included in Figure 3 but available in Table S5. There were no differences between left and right subcortical hemispheres for significant results in Figure 3 apart from the pallidum (Figures S5 and S6). We analyzed each microstructure region for gFA and gMD latent factors. Areas in the association fiber and thalamic regions such as posterior and anterior thalamic radiation, superior and inferior longitudinal fasciculus, and inferior fronto‐occipital fasciculus were driving associations between IDH taking medications compared to normotensives for both gFA and gMD (Figures S7 and S8).

### 3.3 Differences in brain volumes between isolated hypertensive and systolic‐diastolic hypertensive participants

**FIGURE 4:**
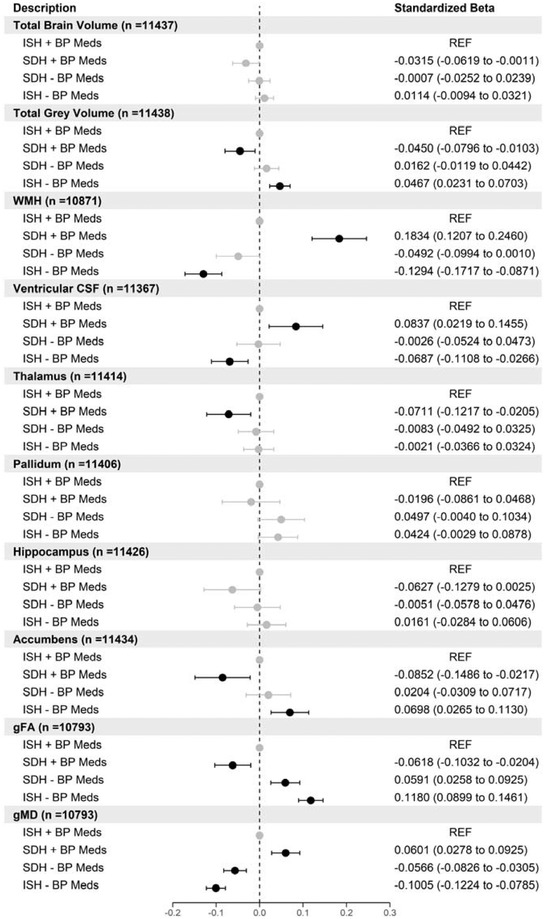
Forest plot showing the association of different brain volumes with isolated systolic hypertension (ISH) versus systolic‐diastolic hypertension (SDH) in participants stratified by blood pressure medication use (reference group ISH taking blood pressure medications). Points in black are statistically significant (FDR *p* < .05) standardized betas (*n* = 11,639)

We investigated whether there were differences in brain volumes between participants with isolated hypertension (either systolic or diastolic) versus those with SDH split by BP medication use. For this analysis, the reference group was those with isolated hypertension and taking BP medications. In Figure 4, we present the results of the analysis comparing ISH and SDH for those brain volumes who showed significant differences between normotensives (not taking BP medication) participants. This was under the assumption if there were no differences between normotensives and hypertensive participants with isolated hypertension then it would be unlikely there would be meaningful differences between isolated and SDH participants. In this analysis, there are four groups. The first group (reference group) contained participants with ISH taking BP medications (*n* = 2616), the second group contained participants with SDH taking BP medications (*n* = 1001), the third group contained participants with SDH not taking BP medications (*n* = 2464), and finally the fourth group containing participants with ISH not taking BP medications (*n* = 5558) (Figure 1).

Participants with SDH and taking BP medications have smaller total gray (*β* = −.05), thalamus (*β* = −.07), accumbens (*β* = −.09), gFA values (*β* = −.06) and larger WMH (*β* = .18), ventricular CSF (*β* = .08), and gMD (*β* = .06) compared with ISH participants taking BP medications. Participants with ISH and not taking BP medications had larger total gray matter (*β* = .05), accumbens (*β* = .07), gFA values (*β* = .11) but lower WHM (*β* = −.13), ventricular CSF (*β* = −.07) and gMD (*β* = −.10). Participants with SDH not taking medications only had larger gFA (*β* = .06) and smaller gMD values (*β* = −.06) compared to the reference group (ISH taking medications). Left and right subcortical regions showed no differences for thalamus but side specificity for pallidum for participants with SDH not taking medications, and accumbens for participants with SDH taking medications (Figures S9 and S10). Analysis of the individual white microstructural regions of the FA and MD overall showed similar patterns to the latent measures for FA and MD in Figure 4. However, there were cases where participants with SDH (with and without medication) had regions that were not significantly different to participants with ISH taking BP medications (Figures S11 and S12).

**FIGURE 5:**
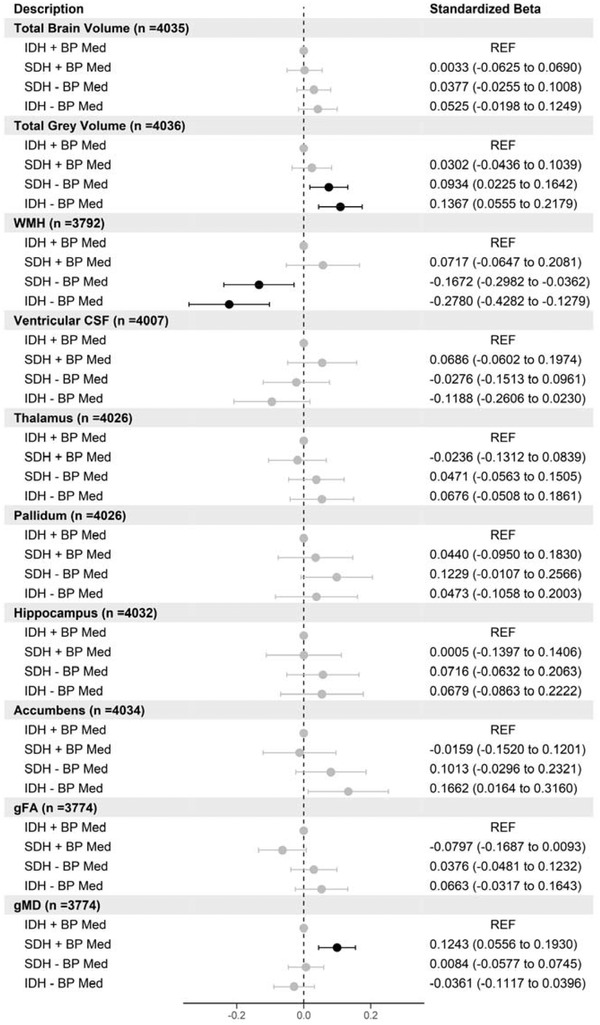
Forest plot showing the association of different brain volumes with isolated diastolic hypertension (IDH) versus systolic‐diastolic hypertension (SDH) in participants stratified by blood pressure medication use (reference group IDH taking blood pressure medications). Points in black are statistically significant (FDR *p* < .05) standardized betas (*n* = 4113)

We then carried out the same analysis but using IDH groups. There were 191 participants with IDH taking BP medications, 1001 participants with SDH taking BP medications, 2464 participants with SDH not taking BP medications, and 457 with IDH not taking medications, respectively. Figure 5 shows that participants with IDH or SDH not taking medications have larger total gray matter volumes (SDH *β* = .09, IDH *β *= .14) and also have smaller WMH volumes (SDH *β* = −.17, IDH *β* = −.28). Participants with SDH taking medications larger gMD values (*β* = .12). There were no side‐specific differences for subcortical regions between any groups (data not shown). There were no differences in white matter microstructural regions for FA but for those with SDH taking medications their white microstructural regions for MD in association and thalamic regions were larger (Figures S13 and S14).

In additional analysis, we repeated all analysis including previously excluded participants with neurological conditions (*n* = 968). Inclusion of these participants in all analysis showed no differences in results (data not shown). Finally, for this current study, we used BP cut‐offs according to the 2018 ESC/ESH Guidelines (Williams et al., 2018); however, the 2017 AHA/ACC guidelines (Whelton et al., 2018) lowered the definition of hypertension to ≥130/≥80 mm Hg. Therefore, we repeated all analyses using the lower 2017 AHA/ACC guidelines. This additional analysis was carried out as if some major differences are shown between these analyses, they could add significant insights over the choice between standard and intensive BP control. These additional analyses showed no significant differences compared to using the higher 2018 ESC/ESH Guidelines (data not shown).

## 4 DISCUSSION

In this largest imaging study to date on this subject, using data from UK Biobank, we found evidence that participants with isolated systolic or diastolic hypertension were associated with smaller total gray matter, larger WMH, and differences between white matter microstructures compared to normotensives. Compared to those with SDH, those with isolated hypertension, particularly ISH had larger total gray matter, smaller WMH, and better white matter integrity. With all analyses, BP medication use was important in these comparisons.

### 4.1 The impact of blood lowering medication on brain volumes

When comparing normotensives versus participants with isolated hypertension, we split all groups by BP medication use. This was firstly to make sure our normotensive group only contained participants with no history of hypertension and secondly for any participants with BP medication it implies a hypertension diagnosis and being controlled by BP medications. It is possible that participants with ISH, ISD, or SDH not taking medications are a mixture of participants with undiagnosed hypertension or who were misclassified due to systematic errors of the BP readings. This could explain why some of the brain volumes for this group were not significantly different from normotensives due to the misclassified participants attenuating the results. However, for WMH and the latent factors of white matter integrity, there were significant differences between normotensives with isolated hypertension regardless of BP medication use, indicating that any undiagnosed participants in this group could be driving differences in WMH and markers of white matter integrity (gFA, gMD). Our results could indicate that antihypertensive therapy is not necessarily likely to lower the impact of long term exposure of elevated BP and resultant smaller brain volumes and greater WHM burden (Kim et al., 2020; Messerli et al., 2021). It is also possible that people with more brain pathology have worse BP and then subsequently are treated with BP medications, which could also explain our results. Disease severity and duration of hypertensive burden could also explain why we found differences between normotensives taking BP medications and smaller or no effects related to isolated hypertensive groups. Those normotensives taking BP medications could be participants with controlled hypertension where hypertension has been controlled by medication and could have a longer time since diagnosis. Although, those with isolated hypertension (with and without BP medications) could contain a mixture of participants with a shorter duration of hypertensive burden and participants with uncontrolled hypertension which could explain a lack of associations between those with isolated hypertensives and true normotensives as well as potential misclassification error.

### 4.2 Isolated systolic hypertension and isolated diastolic hypertension

In this study, we observed that there were more differences across brain measures for those with ISH compared to participants with IDH. Despite this, similar patterns in brain outcomes were observed in both diastolic and systolic hypertension particularly those taking BP medications. It is not surprising the lower number of participants with IDH mainly due to the prevalence being highest in younger participants (Franklin et al., 2001; Sagie et al., 1993). Only, 1274 participants were younger than 50 years out of 29,775 participants used in this study.

Although there are no specific studies focusing on IDH with brain volumes, there are examples of studies focusing on systolic and diastolic BP and brain volumes. Power et al. (2016) showed that higher diastolic BP readings measured 15 and 24 years before imaging were associated with smaller parietal, temporal, and occipital brain regions, whereas higher systolic BP 15 years before imaging was associated with smaller brain volume regions. Although, the BP measures measured at the same time as the MRI measures resulted in no significant relationships. More recently, Lane et al. (2019) using 1946 British birth cohort found that high and increasing BP (either systolic and diastolic) from early adulthood into midlife appeared to be associated with increased WMH and smaller total brain volumes at 69−71 years old. The issue with previous studies is it is difficult to disentangle the impact of each BP measure on the other to determine whether each one in isolation is related to smaller brain volumes. This is where our current study is a vast improvement by examining the individual components of ISH and IDH to brain measures. In this study, we found no significant differences in associations when we used the 2018 ESC/ESH Guidelines (Williams et al., 2018) or the 2017 AHA/ACC guidelines (Whelton et al., 2018) for defining hypertension when comparing brain measures. This indicates that lower thresholds for BP are still associated with smaller brain measures and larger white matter structures, which has implications on when standard and intensive BP control should be assessed and monitored in relation to brain health and future dementia risk.

### 4.3 Isolated hypertension and the brain

Our study using UK Biobank is in agreement with the majority of studies showing associations between hypertension and brain volumes (Cox et al., 2019; d’Arbeloff et al., 2019; Ferguson et al., 2020; Lane et al., 2019; Launer et al., 2015; Lyall et al., 2016; Wiseman et al., 2004). However, unlike our study, none focuses specifically on isolated hypertension and multiple brain measures. In particular, Cox et al. (2019) using an earlier subset of UK Biobank imaging cohort (*n* = 9722) showed that individual risk factors such as hypertension and combinations of vascular risk factors were associated with poorer brain health across global brain measures, subcortical, white matter and white matter macrostructure and microstructures. However, they only used information on self‐reported hypertension, so there was no information on the specific subtype of hypertension. In this study, we found few associations between subcortical volumes with participants with isolated hypertension. This could be down to lack of power to detect very small effect sizes which have been observed in previous studies using UK Biobank (Cox et al., 2019; Newby et al., 2021). Although we did not find significant associations for isolated hypertension, we did show that there were differences between right and left sides for some subcortical brain measures in those with controlled hypertension. This supports evidence of side specificity of brain measures related to dementia and cognitive decline in those controlled hypertension (Toga & Thompson, 2003).

Hypertension causes multiple pathological alterations in the brain that damage brain structures which are associated with cognitive decline and increased dementia risk (Ungvari et al., 2021). In this study, we showed that isolated hypertension is associated with poorer global brain measures and greater white matter pathologies which are also associated with cognitive decline. Therefore, isolated hypertension although appears to less detrimental than SDH is likely to cause similar pathology and hence affects cognitive decline.

### 4.4 Isolated hypertension and SDH

We found that those with ISH were associated with larger gray brain measures and smaller white matter micro and macrostructures compared with SDH however only in those taking BP medications. This could indicate different disease phenotypes arising from different hemodynamic mechanisms between isolated and SDH. ISH has been shown to result from large artery stiffness in contrast to SDH, which mostly affects small arteries, arterioles, and increased peripheral vascular resistance (McEniery et al., 2005; Schiffrin, 2004). The combined effect of both these processes and sustained rise in BP could be sufficient to exceed the upper limit of cerebral blood flow autoregulation, which could explain why SDH have smaller brain measures.

### 4.5 Strengths and limitations of the study

UK Biobank to date has the largest single‐protocol imaging study with more than 50,000 participants from the general population imaged so far. Information from participants contain detailed information on health and lifestyle allowing for in‐depth analysis relating to health such as isolated hypertension with brain health. UK Biobank also provides image‐derived phenotypes of a variety of brain measures which allow an assessment of isolated hypertension across an array of brain measures to aid further understanding between the link between brain health and disease.

This study is cross‐sectional; therefore, we cannot define temporal associations for causal inference. It could be possible that some participants with ISH in this study could previously have had either IDH or ISH, and this could be inflating some of the differences seen in our results. For the comparison between isolated hypertension versus SDH, we used participants with isolated hypertension taking BP medications as the reference group. As the data are cross‐sectional, it is possible that some participants could previously have previous IDH or SDH but medication use failed to reduce either systolic or diastolic BP measures. Therefore, there must be some caution in the interpretation of these results. As ISH can occur de novo or from IDH or SDH, future work will examine baseline hypertension and see how these groups from baseline to imaging visit influence brain health. Furthermore, the impact of isolated hypertension on cognition can be assessed when more imaging becomes available to establish the link hypertension‐correlated brain alterations with cognitive assessments. It has shown that 59% of ISH can develop de novo and this could be much higher with increasing age (Franklin et al., 2005); therefore, those with greater hypertensive burden could be driving our results. For this study, we split our groups by BP medication use. This information was self‐reported, so it is possible that there are misclassifications due to recall biases, which could attenuate results. For the IDH analysis in particular, low sample sizes in these groups as well as difference in samples sizes between all of the groups analyzed which may limit our findings. The relationships between isolated hypertension and brain structure are detectable even in these relatively healthy participants in UK Biobank; therefore, it is possible that the effects seen will be more pronounced in a more representative population sample. Despite this, the population is predominately Caucasian, which could restrict the generalizability to other ethnicities. We did remove people with neurological and neurodegenerative disorders and it is possible due to recall bias that some participants may have some of these disorders, which could potentially be driving the results.

## 5 CONCLUSIONS

Participants with isolated hypertension were associated with poorer brain health across gray and white matter compared to participants with normal BP, but medication use was important. Additionally, those with SDH compared to ISH also had poorer brain health. White matter macrostructures and microstructures showed the strongest differences between all groups in all analysis emphasizing the importance of markers of cerebrovascular health. This study supports the importance of maintaining a healthy BP with regard to the preservation of brain and cognitive health in later life. Furthermore, the implications of our study do support a potential need for specific guidelines for risk management for isolated hypertension alongside SDH particularly regarding current and future brain health. Further research needs to increase understanding of the underlying mechanisms involved in hypertension‐related brain changes between the different subtypes, and how and when this influences cognitive decline and future dementia risk.

## CONFLICT OF INTEREST

Alejo J. Nevado‐Holgado has received funding from Janssen Pharmaceuticals, GlaxoSmithKline and Ono Pharma. All other authors declare no conflict of interest.

## AUTHOR CONTRIBUTIONS

Danielle Newby and Lenore J. Launer conceived and designed the study with input and all other authors. Danielle Newby wrote the manuscript with input from Laura Winchester, Alejo J. Nevado‐Holgado, and Lenore J. Launer. All other authors reviewed the final manuscript.

### PEER REVIEW

The peer review history for this article is available at https://publons.com/publon/10.1002/brb3.2525.

## Supporting information

Supporting Information

## Data Availability

UK Biobank is an open access resource available to verified researchers upon application (http://www.ukbiobank.ac.uk/). Analysis syntax is available on request.
