# Supplementary material for: The relationship between isolated hypertension with brain volumes in UK Biobank
Source: Brain Behav. 2022 Apr 1;12(5):e2525. doi: 10.1002/brb3.2525 (PMC9120723; doi:10.1002/brb3.2525)
Supplement: Supplementary file 1 — Supporting Information [file BRB3-12-e2525-s001.docx]

**Supporting information**

**PAPER TITLE:** The relationship between Isolated hypertension with Brain Volumes in UK Biobank

**Table S1. Self-reported health variables codes used for exclusion criteria on initial population**

| **Condition** | **Code** |
| --- | --- |
|  | *Self reported Illness (Field ID 20002)* |
| Dementia or Alzheimer’s disease | 1263 |
| Parkinson’s disease | 1262 |
| Chronic degenerative neurological | 1258 |
| Guillain-Barré syndrome | 1256 |
| Multiple Sclerosis | 1261 |
| Other demyelinating disease | 1397 |
| Stroke or ischaemic stroke | 1081 |
| Brain cancer | 1032 |
| Brain haemorrhage | 1491 |
| Brain/intracranial abscess | 1245 |
| Cerebral aneurysm | 1425 |
| Cerebral palsy | 1433 |
| Encephalitis | 1246 |
| Epilepsy | 1264 |
| Head injury | 1266 |
| Infections of the nervous system | 1244 |
| Ischaemic stroke | 1583 |
| Meningeal cancer | 1031 |
| Meningioma (benign) | 1659 |
| Meningitis | 1247 |
| Motor Neuron Disease | 1259 |
| Neurological injury/trauma | 1240 |
| Spina bifida | 1524 |
| Subdural haematoma | 1083 |
| Subarachnoid haemorrhage | 1086 |
| Transient ischaemic attack | 1082 |

**Table S2. UKBiobank Field codes for all variables used in manuscript**

| **Variable** | **Code** |
| --- | --- |
| *Hypertension variables* |  |
| systolic blood pressure | 4080 |
| diastolic blood pressure | 4079 |
| BP medications | 6177/6153 |
| *Neuroimaging* |  |
| Total Brain Volume (TBV) | 25010 |
| Total Grey Matter (TGM) | 25006 |
| Total White Matter (WM) | 25008 |
| White matter hyperintensities (WMH) | 25781 |
| Ventricular CSF | 25004 |
| Hippocampus (L+R) | 25019/20 |
| Thalamus (L+R) | 25011/12 |
| Caudate (L+R) | 25013/14 |
| Putamen (L+R) | 25015/16 |
| Pallidum (L+R) | 25017/18 |
| Amygdala (L+R) | 25021/22 |
| Accumbens (L+R) | 25023/24 |
| gFA (fractional anisotropy) | 25488-25514 |
| gMD (mean diffusivity) | 25515-25541 |
| *Confounding Variables* |  |
| Education | 6138 |
| Townsend deprivation index at recruitment | 189 |
| Smoking Status | 20116 |
| Gender | 31 |
| Age at Assessment | 21300 |
| Assessment Centre | 54 |
| BMI | 21001 |
| Ethnicity | 21000 |
| Diabetes | Field ID 20002: Code 1220, 1222, 1223 & Field IDs 130708, 130708, 130710, 130712, 130712, 6177, 6153 |
| High Cholesterol | Field ID 20002, Code 1473 & Field IDs 6177, 6153 |
| Head size | 25000 |
| Scanner Position X | 25756 |
| Scanner Position Y | 25757 |
| Scanner Position Z | 25758 |
| Scanner Position | 25759 |

**Field IDs obtained only for imaging visit apart from Ethnicity where baseline visit information was also used.**

**Table S3: Characteristics of UK Biobank participants at imaging visit included stratified by hypertensive state and BP medication usage (compliments Table 1 in main manuscript)**

| Description | Normotensive - BP Meds (n=14584) | Normotensive + BP Meds (n=2904) | IDH - BP Meds (n = 457) | IDH + BP Meds (n = 191) | ISH - BP Meds (n = 5558) | ISH + BP Meds (n = 2616) | Systolic-diastolic hypertensive - BP Meds (n = 2464) | Systolic-diastolic hypertensive + BP Meds (n = 1001) | N |
| --- | --- | --- | --- | --- | --- | --- | --- | --- | --- |
| Demographics |  |  |  |  |  |  |  |  |  |
| Age years (mean (SD)) | 61.3 (7.42) | 65.8 (6.74) | 59.2 (7.20) | 62.2 (7.27) | 65.8 (6.83) | 68.2 (6.05) | 62.8 (7.26) | 65.0 (7.10) | 29775 |
| Gender (Male N (%)) | 5635 (38.6%) | 1658 (57.1%) | 240 (52.5%) | 116 (60.7%) | 2795 (50.3%) | 1594 (60.9%) | 1446 (58.7%) | 643 (64.2%) |  |
| Ethnicity (White N (%)) | 14095 (96.9%) | 2789 (96.3%) | 426 (93.6%) | 183 (95.8%) | 5431 (97.9%) | 2536 (97.2%) | 2390 (97.3%) | 960 (96.2%) |  |
| Education – Degree N (%) | 7614 (52.6%) | 1291 (44.6%) | 238 (52.4%) | 77 (40.5%) | 2542 (46.3%) | 1084 (41.7%) | 1161 (47.8%) | 423 (42.5%) |  |
| Townsend Deprivation Index Decile (mean (SD)) | 5.55 (2.87) | 5.54 (2.89) | 5.61 (2.93) | 5.74 (2.90) | 5.28 (2.84) | 5.38 (2.82) | 5.39 (2.85) | 5.45 (2.88) | 29750 |
| Assessment Centre N (%) |  |  |  |  |  |  |  |  |  |
| Cheadle | 10136 (69.5%) | 2019 (69.5%) | 328 (71.8%) | 152 (79.6%) | 3477 (62.6%) | 1651 (63.1%) | 1646 (66.8%) | 690 (68.9%) | 29775 |
| Reading | 1966 (13.5%) | 460 (15.8%) | 46 (10.1%) | 19 (9.95%) | 580 (10.4%) | 288 (11.0%) | 175 (7.10%) | 74 (7.39%) |  |
| Newcastle | 2482 (17.0%) | 425 (14.6%) | 83 (18.2%) | 20 (10.5%) | 1501 (27.0%) | 677 (25.9%) | 643 (26.1%) | 237 (23.7%) |  |
| BMI Kg/m2 (mean (SD)) | 25.5 (3.89) | 28.4 (4.74) | 27.8 (4.54) | 30.0 (5.83) | 26.4 (4.06) | 28.2 (4.57) | 27.7 (4.43) | 28.5 (4.54) | 29775 |
| Smoking Status (Ever/Current (N (%))) | 5131 (35.4%) | 1216 (42.1%) | 143 (31.6%) | 75 (39.5%) | 2026 (36.9%) | 1152 (44.2%) | 856 (35.3%) | 415 (41.5%) |  |
| Diastolic Blood Pressure mm Hg (mean (SD)) | 73.6 (7.71) | 74.5 (7.81) | 93.0 (3.37) | 92.8 (3.12) | 80.2 (6.43) | 79.4 (6.63) | 95.8 (5.32) | 95.9 (5.33) | 29775 |
| Systolic Blood Pressure mm Hg (mean (SD)) | 123 (10.4) | 127 (9.20) | 133 (5.59) | 134 (4.61) | 151 (10.3) | 153 (11.2) | 159 (14.3) | 160 (14.2) | 29775 |
| Taking Blood pressure Medications (N (%)) | 0 (0.00%) | 2904 (100%) | 0 (0.00%) | 191 (100%) | 0 (0.00%) | 2616 (100%) | 0 (0.00%) | 1001 (100%) |  |
| Hypercholesterolaemia (N (%)) | 1937 (13.3%) | 1638 (56.4%) | 56 (12.3%) | 80 (41.9%) | 1107 (19.9%) | 1508 (57.6%) | 421 (17.1%) | 446 (44.6%) |  |
| Diabetes (N (%)) | 377 (2.59%) | 437 (15.0%) | 13 (2.84%) | 17 (8.90%) | 217 (3.90%) | 383 (14.6%) | 79 (3.21%) | 101 (10.1%) |  |
| Brain Volumes |  |  |  |  |  |  |  |  |  |
| Total Brain Volume mm3 (mean (SD)) | 1165103 (110222) | 1159529 (110213) | 1186162 (112282) | 1179820 (109123) | 1155204 (112713) | 1154815 (108850) | 1175816 (113025) | 1170725 (112440) | 29768 |
| Grey Matter mm3 (mean (SD)) | 620288 (54719) | 608717 (55778) | 630585 (55922) | 618803 (54442) | 610737 (55926) | 604946 (54607) | 621133 (55722) | 614043 (56811) | 29771 |
| WMH mm3 (mean (SD)) | 3330 (3752) | 5728 (5499) | 3504 (4008) | 5522 (5486) | 5007 (4927) | 6663 (6067) | 4647 (4812) | 6708 (6196) | 28357 |
| *g*FA units *M* (SD) | 0.09 (0.52) | -0.09 (0.58) | 0.09 (0.53) | -0.03 (0.59) | -0.03 (0.55) | -0.18 (0.60) | -0.02 (0.57) | -0.17 (0.59) | 28025 |
| *g*MD units *M* (SD) | -0.09 (0.41) | 0.10 (0.48) | -0.12 (0.43) | -0.01 (0.48) | 0.04 (0.45) | 0.20 (0.50) | 0.01 (0.46) | 0.18 (0.49) | 28025 |
| Ventricular CSF mm3 (mean (SD)) | 33007 (14581) | 40100 (17166) | 32003 (14075) | 36986 (15926) | 37534 (15814) | 42139 (17473) | 36554 (15753) | 40717 (17591) | 29636 |
| Hippocampus mm3 (mean (SD)) | 3874 (423) | 3801 (438) | 3922 (421) | 3870 (436) | 3818 (437) | 3775 (447) | 3881 (451) | 3824 (451) | 29739 |
| Accumbens mm3 (mean (SD)) | 457 (103) | 420 (104) | 472 (102) | 444 (112) | 433 (103) | 411 (105) | 449 (105) | 424 (104) | 29760 |
| Amygdala mm3 (mean (SD)) | 1246 (215) | 1256 (216) | 1257 (224) | 1248 (205) | 1248 (215) | 1249 (223) | 1264 (219) | 1252 (217) | 29755 |
| Pallidum mm3 (mean (SD)) | 1783 (213) | 1764 (231) | 1796 (218) | 1780 (224) | 1775 (228) | 1757 (235) | 1801 (229) | 1778 (239) | 29706 |
| Putamen mm3 (mean (SD)) | 4824 (557) | 4764 (573) | 4923 (547) | 4938 (605) | 4765 (569) | 4731 (583) | 4858 (595) | 4841 (589) | 29733 |
| Caudate mm3 (mean (SD)) | 3472 (409) | 3472 (429) | 3518 (414) | 3512 (402) | 3455 (431) | 3465 (408) | 3506 (433) | 3521 (439) | 29730 |
| Thalamus mm3 (mean (SD)) | 7720 (723) | 7585 (707) | 7848 (735) | 7729 (673) | 7600 (729) | 7544 (719) | 7737 (743) | 7634 (715) | 29711 |

Normotensive: diastolic blood pressure BP <90 mm Hg and systolic BP <140 mm Hg; Isolated diastolic hypertensive (IDH): diastolic blood pressure BP ≥90 mm Hg and systolic BP < 140 mm Hg; Isolated systolic hypertensive (ISH): systolic BP ≥140 mm Hg and diastolic BP < 90 mm Hg; systolic-diastolic hypertension (SDH): diastolic blood pressure BP ≥90 mm Hg and systolic BP ≥140 mm Hg. BP medication use was obtained via self report.

**Table S4. Association between isolated systolic hypertension stratified by medication use with brain volumes for non-significant results compared to normotensives**

| **Brain Volume** | **Group** | **Standardized β** | **95% CI** | | **P** |
| --- | --- | --- | --- | --- | --- |
|  |  |  | **Lower** | **Upper** |  |
| Caudate (n =25253) | Normotensive - BP Meds | REF | REF | REF | REF |
|  | Normotensive + BP Meds | 0.0135 | -0.0221 | 0.0491 | 0.4781 |
|  | ISH - BP Meds | 0.0068 | -0.0199 | 0.0334 | 0.6179 |
|  | ISH + BP Meds | 0.0264 | -0.0113 | 0.0641 | 0.2451 |
| Putamen (n =25253) | Normotensive - BP Meds | REF | REF | REF | REF |
|  | Normotensive + BP Meds | -0.0169 | -0.0495 | 0.0158 | 0.4479 |
|  | ISH - BP Meds | 0.0221 | -0.0023 | 0.0465 | 0.1451 |
|  | ISH + BP Meds | 0.018 | -0.0165 | 0.0526 | 0.4479 |
| Amygdala (n =25270) | Normotensive - BP Meds | REF | REF | REF | REF |
|  | Normotensive + BP Meds | -0.0425 | -0.0822 | -0.0028 | 0.0685 |
|  | ISH - BP Meds | -0.0099 | -0.0395 | 0.0198 | 0.5916 |
|  | ISH + BP Meds | -0.0467 | -0.0886 | -0.0047 | 0.0673 |

**Standardized betas, 95% CI and P-values are reported from regression models where hypertension status are regressed onto MRI measures adjusted for age, sex, sex*age, education, ethnicity, assessment centre, body mass index (BMI), smoking status, diabetes, deprivation and hyperlipidemia. position MRI confounds and head size. For brain sub cortical volumes, negative values indicate smaller volumes for each group compared to reference level (Normotensive). ISH: isolated systolic hypertension defined as systolic ≥140mmHg but diastolic bp < 90 mmHg. The numbers for the comparisons were normotensive (n = 14584), normotensive taking BP medications (n = 2904), individuals with ISH taking BP medications (n = 2616) ISH not taking BP medications (n = 5558).**

**Figure S1. Association between isolated systolic hypertension stratified by medication use with left side of brain sub cortical volumes. Points in black are statistically significant (FDR p value < 0.05) standardized betas**


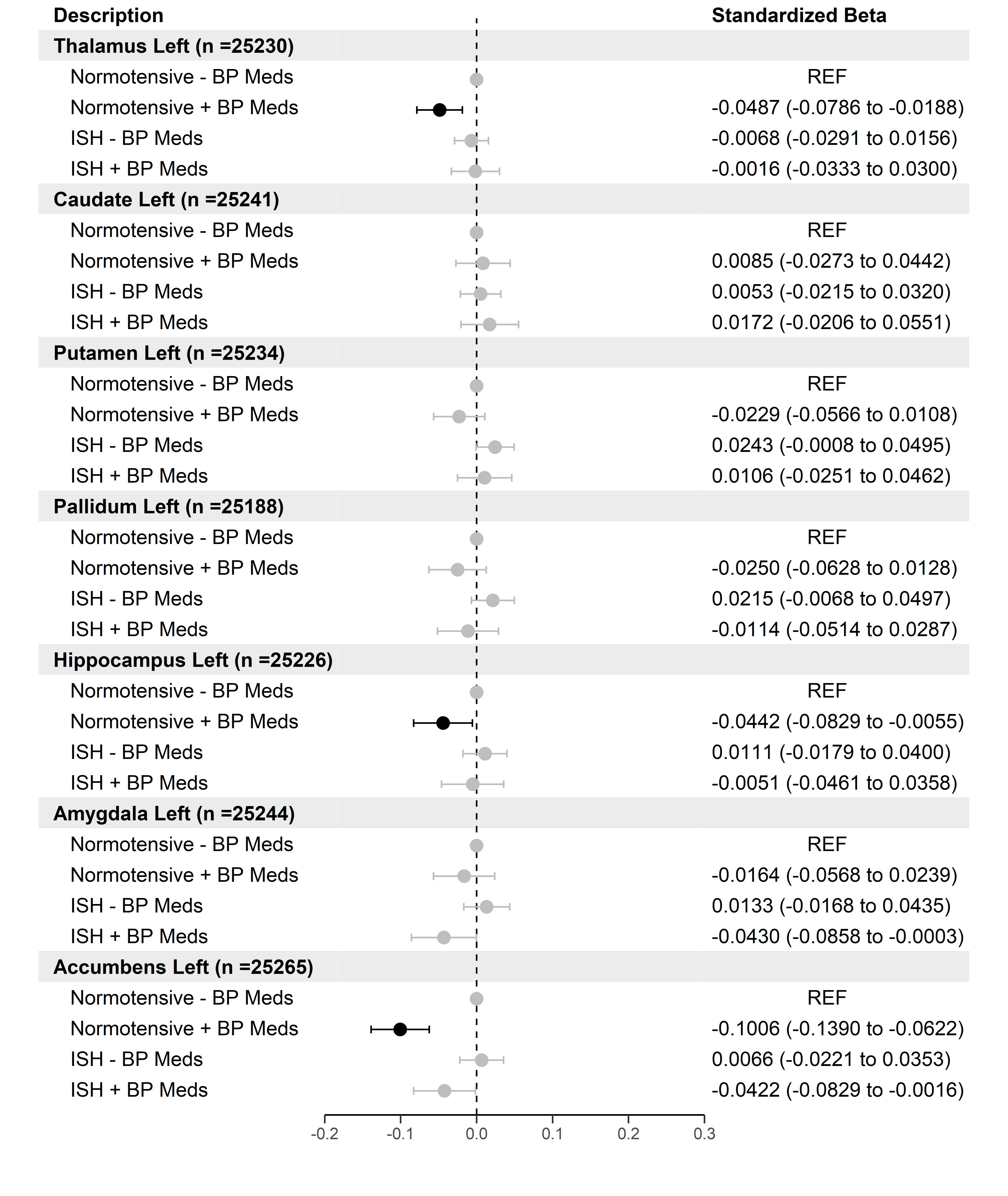


**Figure S2. Association between isolated systolic hypertension stratified by medication use with right side of brain sub cortical volumes. Points in black are statistically significant (FDR p value < 0.05) standardized betas**


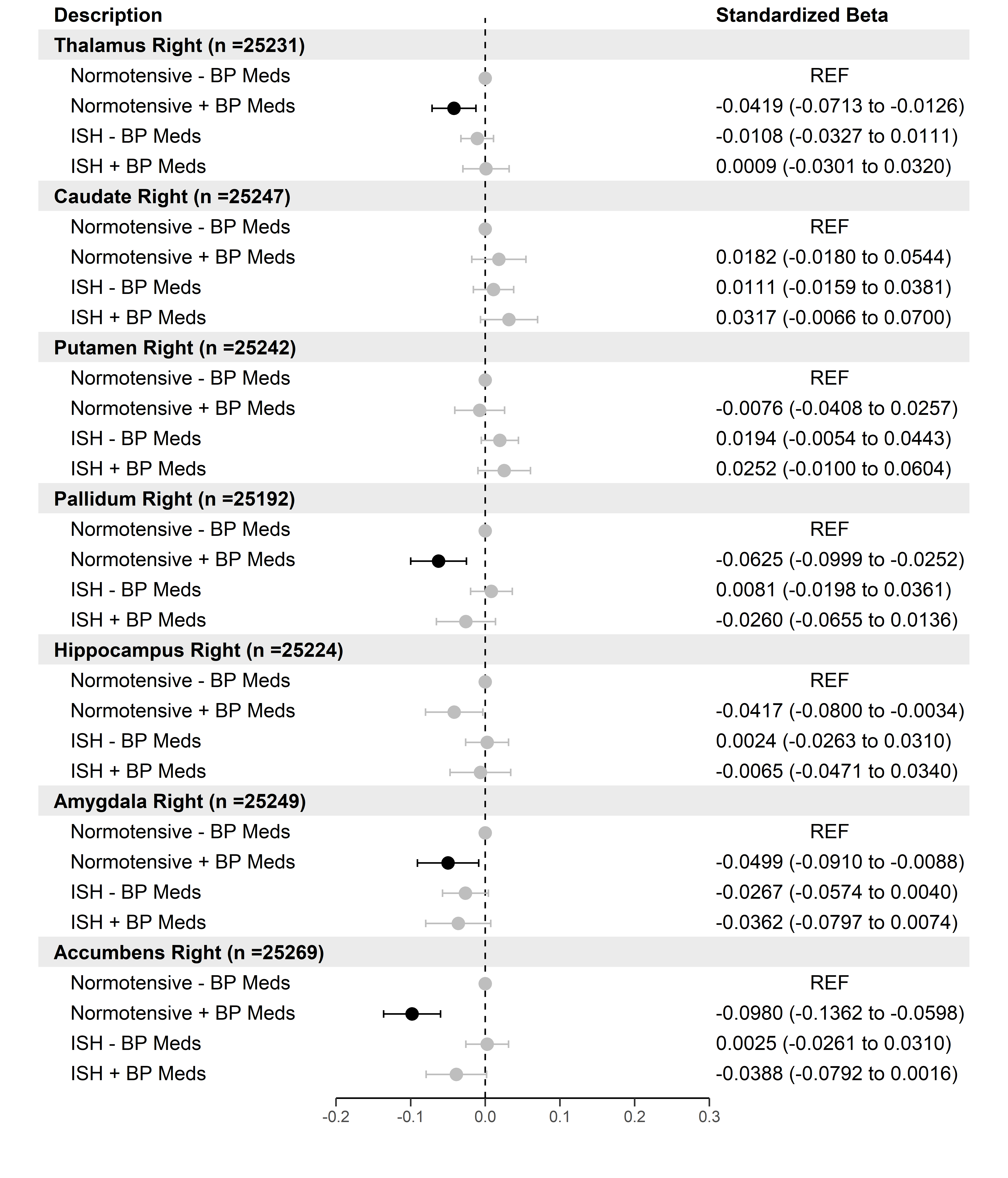

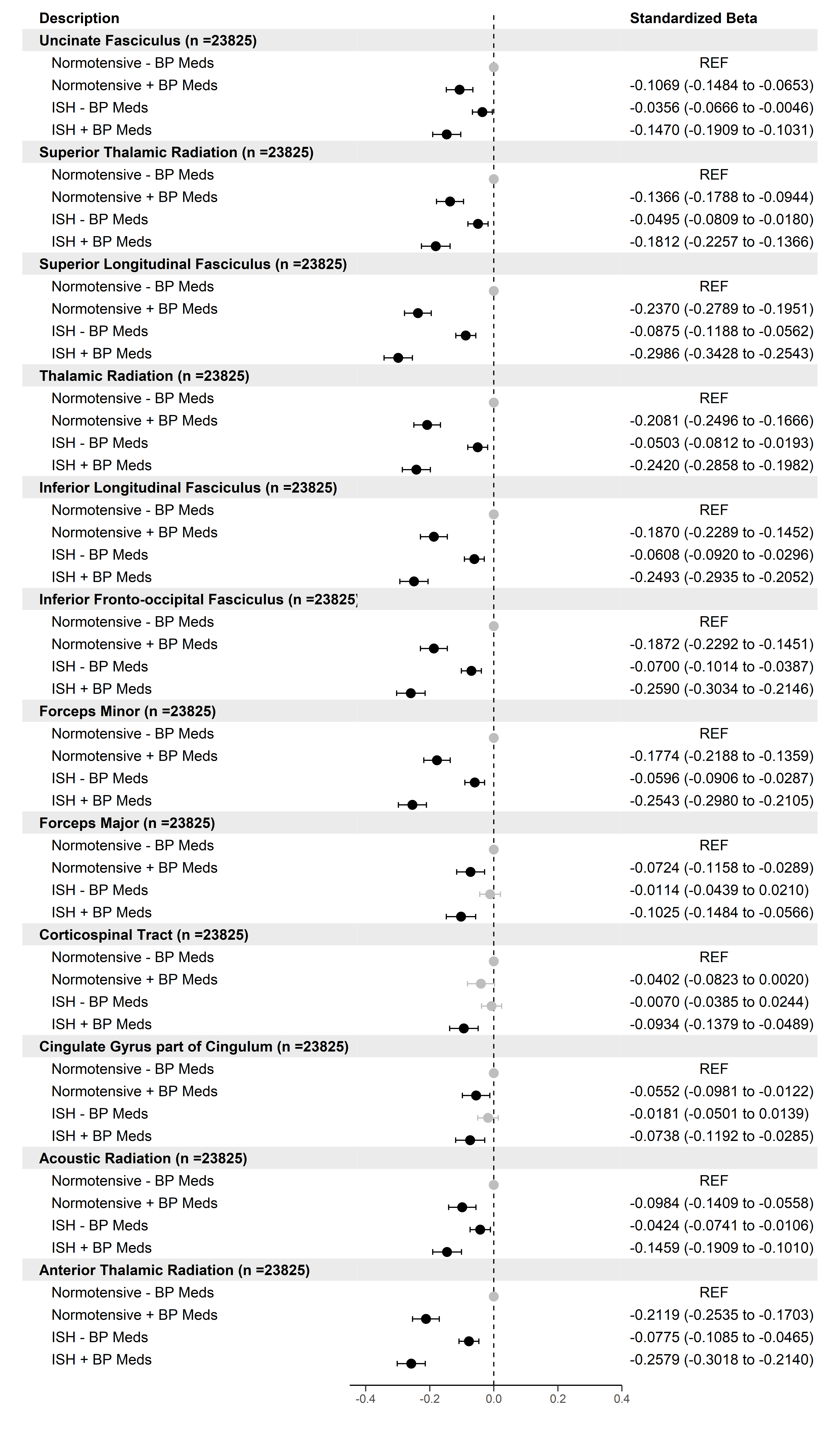


**Figure S3. Association between isolated systolic hypertension stratified by medication use with brain microstructural white matter volumes for fractional anisotropy. Points in black are statistically significant (FDR p value < 0.05) standardized betas**


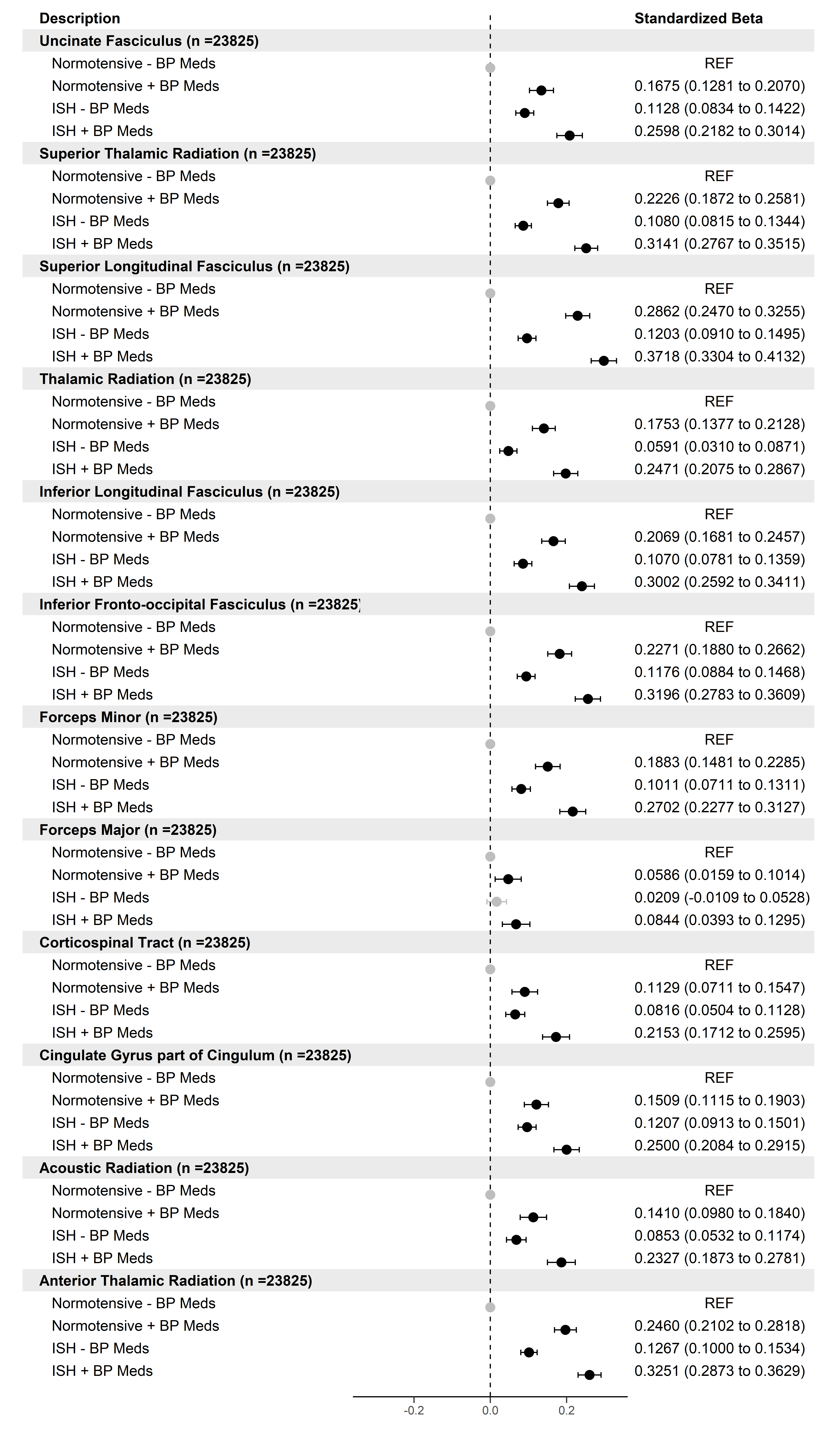


**Figure S4. Association between isolated systolic hypertension stratified by medication use with brain microstructural white matter volumes for mean diffusivity. Points in black are statistically significant (FDR p value < 0.05) standardized betas**

**Table S5. Association between isolated diastolic hypertension stratified by medication use with brain volumes for non-significant results**

| **Brain Volume** | **Group** | **Standardized β** | **95% CI** | | **P** |
| --- | --- | --- | --- | --- | --- |
|  |  |  | **Lower** | **Upper** |  |
| Caudate (n =17861) | Normotensive - BP Meds | REF | REF | REF | REF |
|  | Normotensive + BP Meds | 0.0107 | -0.0259 | 0.0474 | 0.5907 |
|  | IDH - BP Meds | 0.0259 | -0.0505 | 0.1024 | 0.5712 |
|  | IDH + BP Meds | 0.0107 | -0.1077 | 0.1291 | 0.8599 |
| Putamen (n =17862) | Normotensive - BP Meds | REF | REF | REF | REF |
|  | Normotensive + BP Meds | -0.0142 | -0.0477 | 0.0193 | 0.5506 |
|  | IDH - BP Meds | 0.0017 | -0.0684 | 0.0717 | 0.9627 |
|  | IDH + BP Meds | 0.1094 | 0.0013 | 0.2176 | 0.109 |
| Amygdala (n =17869) | Normotensive - BP Meds | REF | REF | REF | REF |
|  | Normotensive + BP Meds | -0.0379 | -0.0789 | 0.0031 | 0.146 |
|  | IDH - BP Meds | -0.0421 | -0.1279 | 0.0437 | 0.4293 |
|  | IDH + BP Meds | -0.1359 | -0.2681 | -0.0036 | 0.1015 |

**Standardized betas, 95% CI and P-values are reported from regression models where hypertension status are regressed onto MRI measures adjusted for age, sex, sex*age, education, ethnicity, assessment centre, body mass index (BMI), smoking status, diabetes, deprivation and hyperlipidemia. position MRI confounds and head size. For brain sub cortical volumes, negative values indicate smaller volumes for each group compared to reference level (normotensives). IDH; isolated diastolic hypertension, IDH is defined as individuals with high diastolic blood pressure (≥90mmHg) but normal systolic blood pressure (<140 mmHg). The numbers for the comparisons were normotensive (n = 14584), normotensive taking BP medications (n = 2904), individuals with IDH taking BP medications (n = 191) IDH + BP medications (n = 457).**

**Figure S5. Association between isolated diastolic hypertension stratified by medication use with left side of brain sub cortical volumes. Points in black are statistically significant (FDR p value < 0.05) standardized betas**


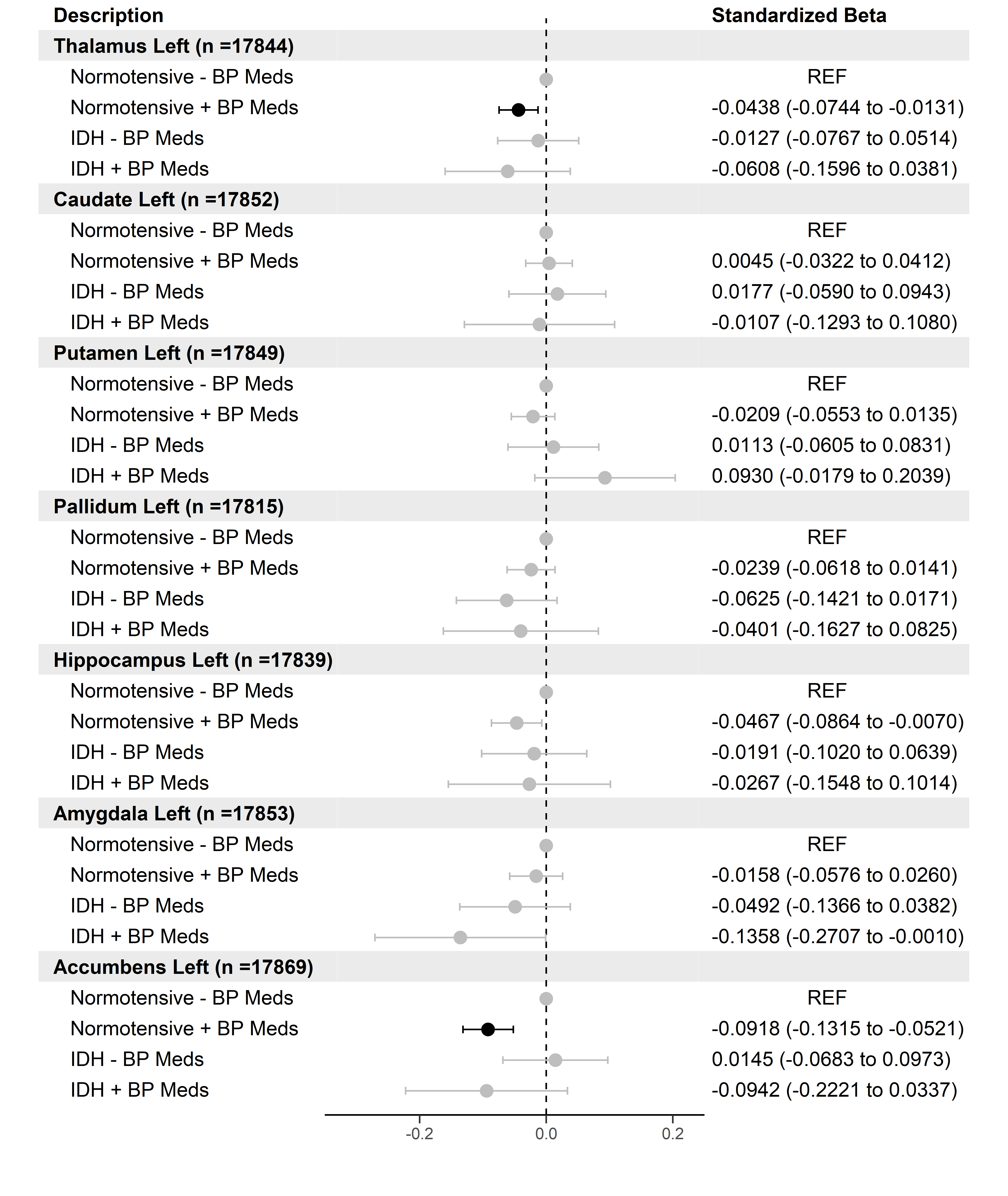


**Figure S6. Association between isolated diastolic hypertension stratified medication use with right side of brain sub cortical volumes. Points in black are statistically significant (FDR p value < 0.05) standardized betas**


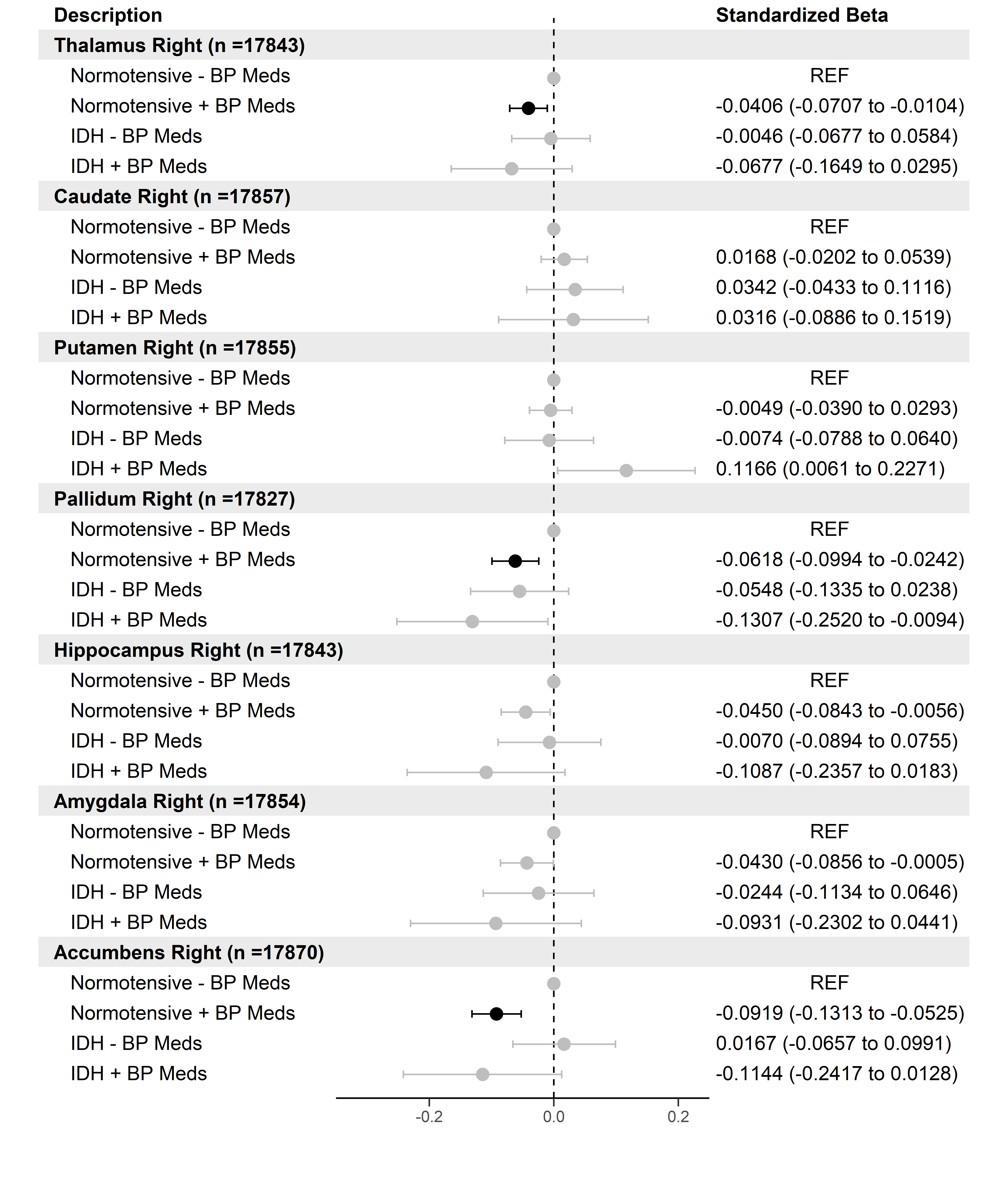

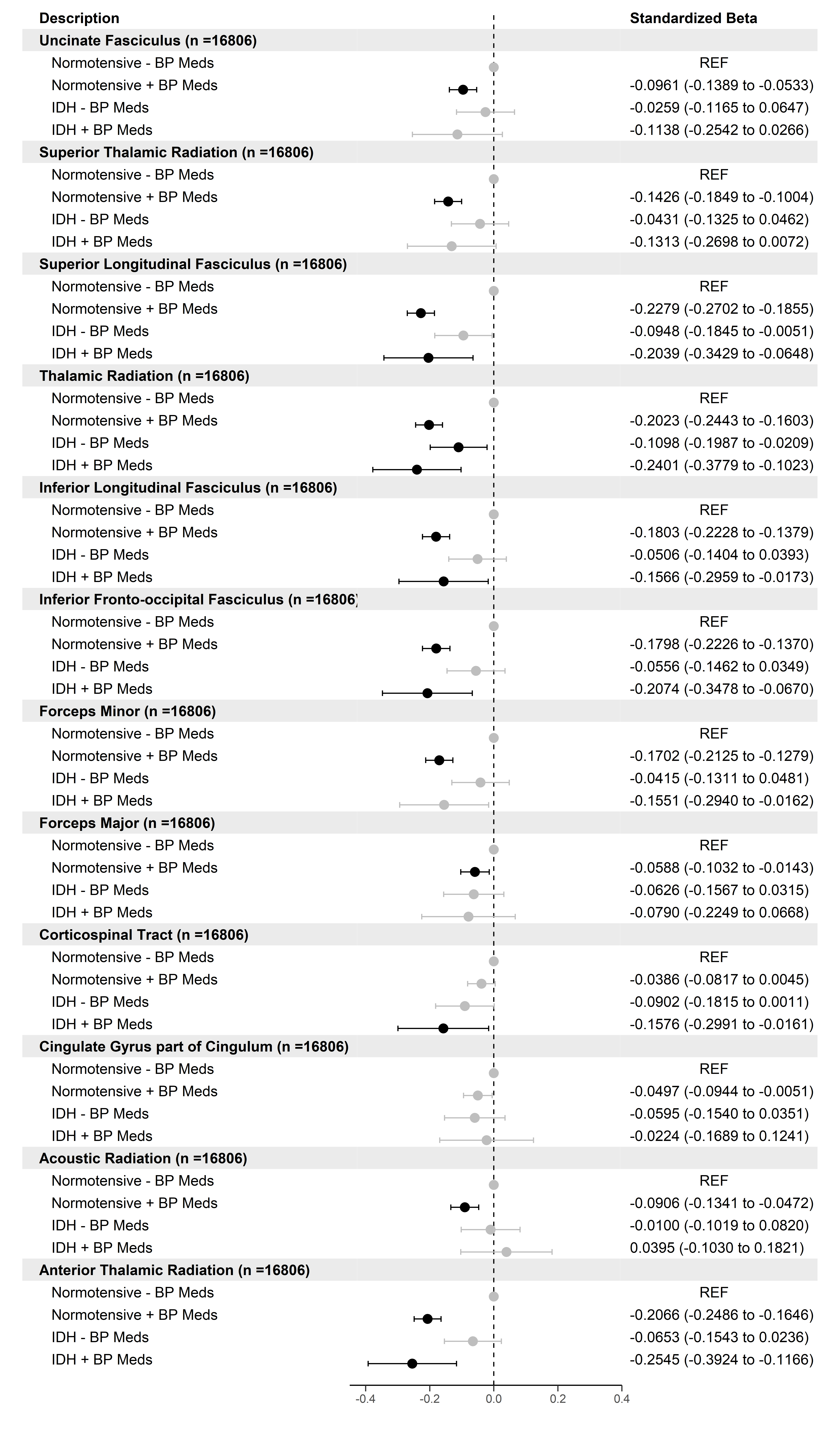


**Figure S7. Association between isolated diastolic hypertension stratified by medication use with brain microstructural white matter volumes for fractional anisotropy stratified by blood pressure medication use. Points in black are statistically significant (FDR p value < 0.05) standardized betas**


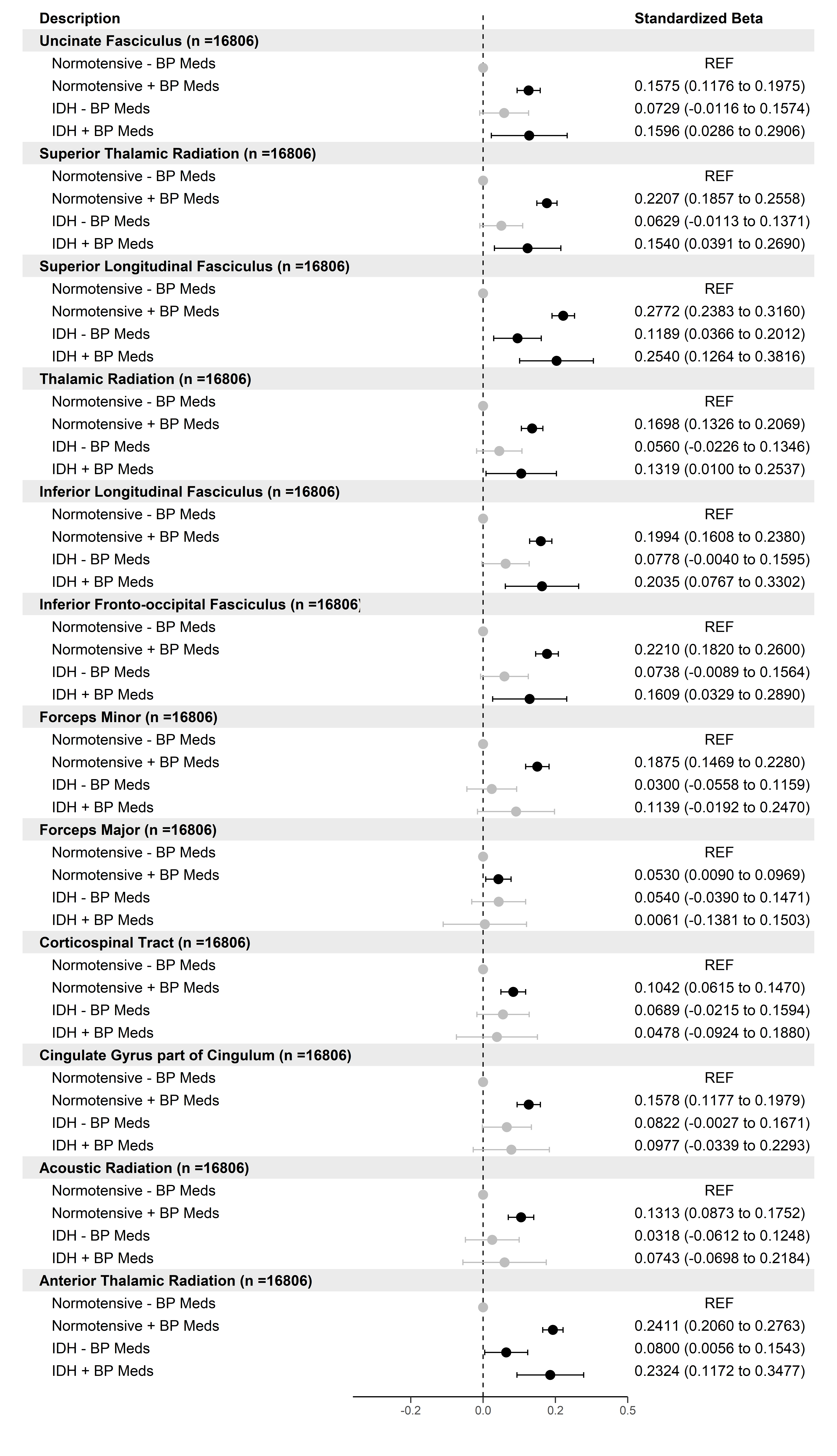


**Figure S8. Association between isolated diastolic hypertension stratified by medication use with brain microstructural white matter volumes for mean diffusivity stratified by blood pressure medication use. Points in black are statistically significant (FDR p value < 0.05) standardized betas**

**Figure S9. Association between isolated systolic hypertension and systo-diastolic hypertension with left sub cortical regions stratified by blood pressure medication use. Points in black are statistically significant (FDR p value < 0.05) standardized betas**


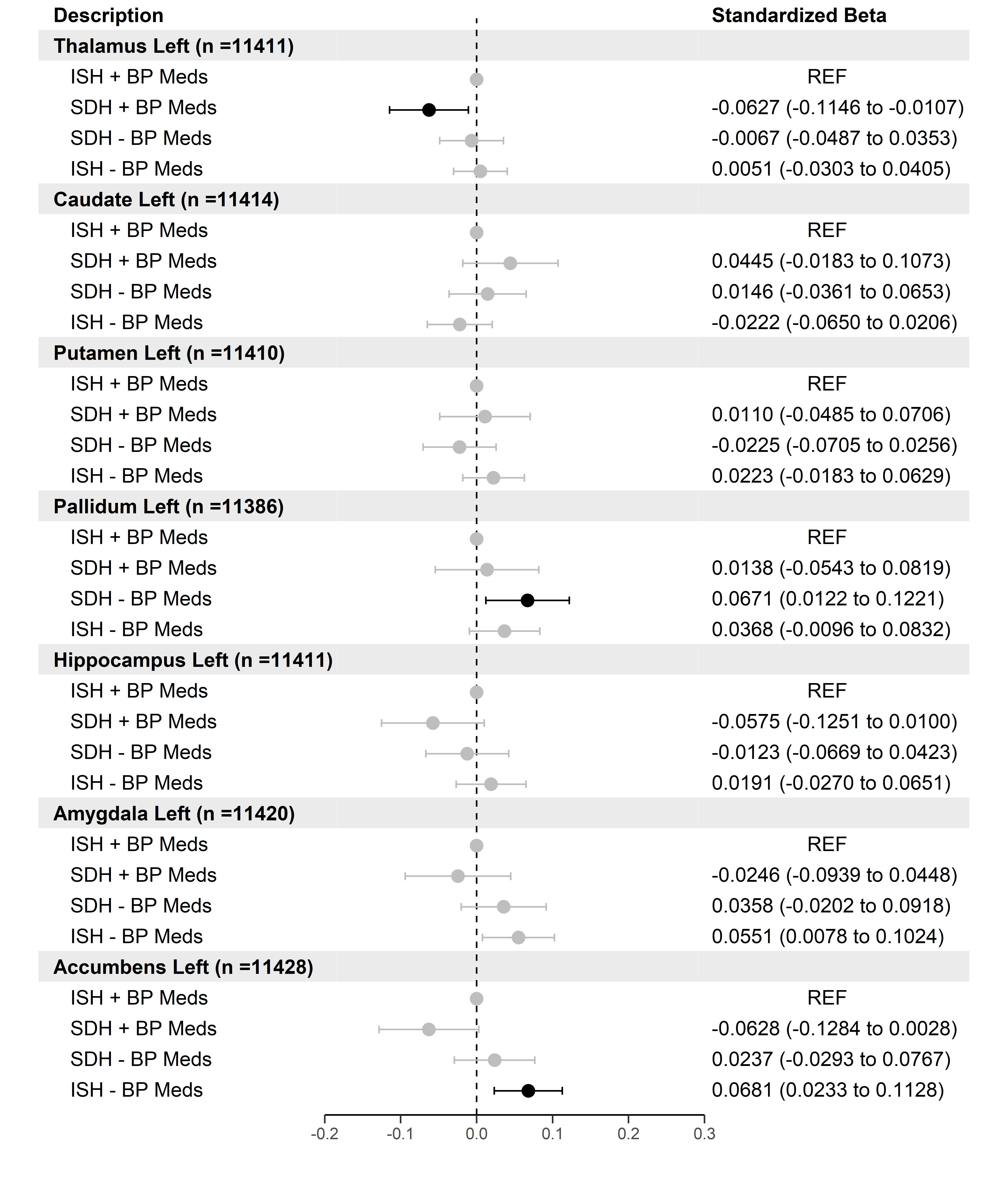


**Figure S10. Association between isolated systolic hypertension and systo-diastolic hypertension with right sub cortical regions diffusivity stratified by blood pressure medication use. Points in black are statistically significant (FDR p value < 0.05) standardized betas**


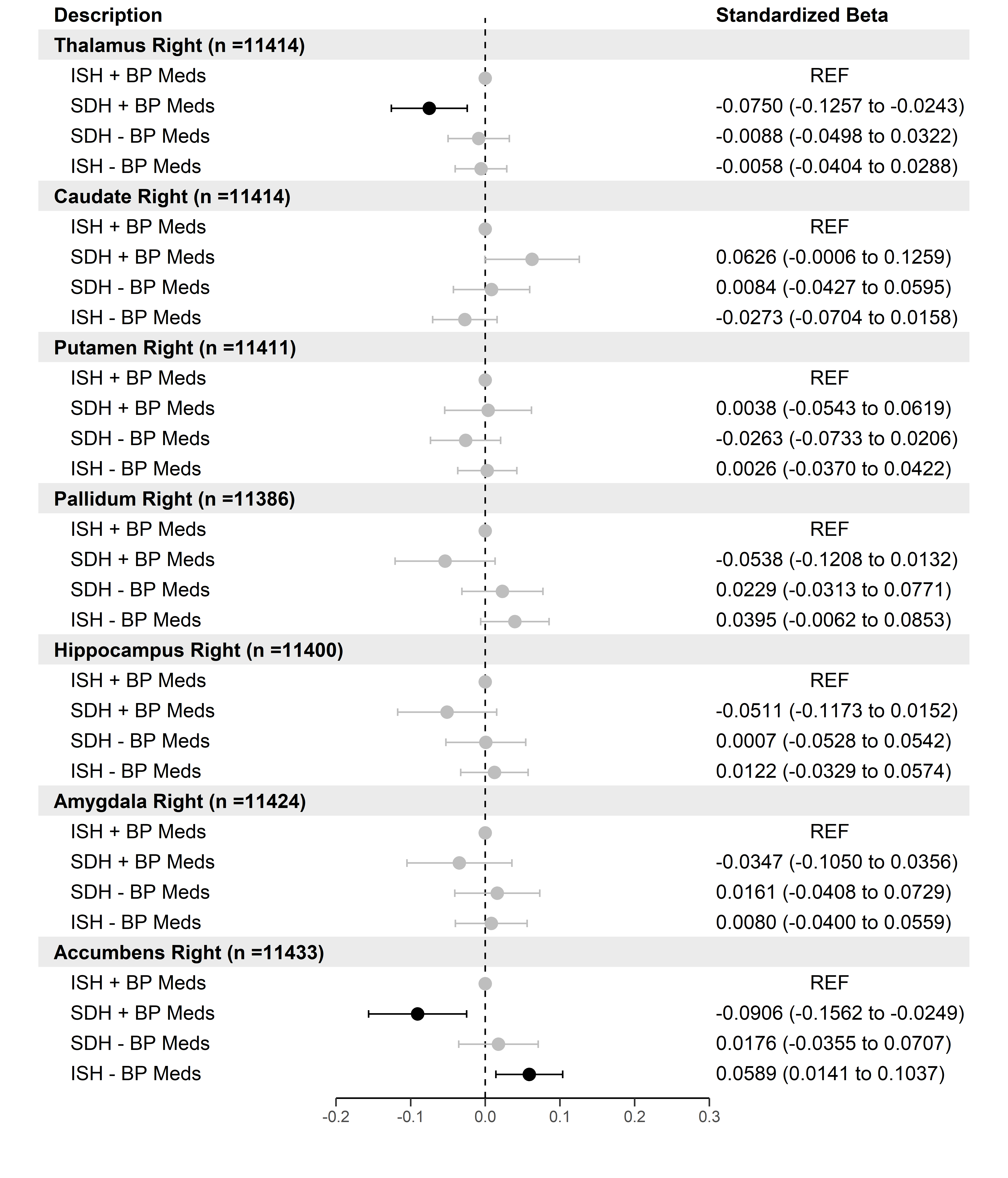

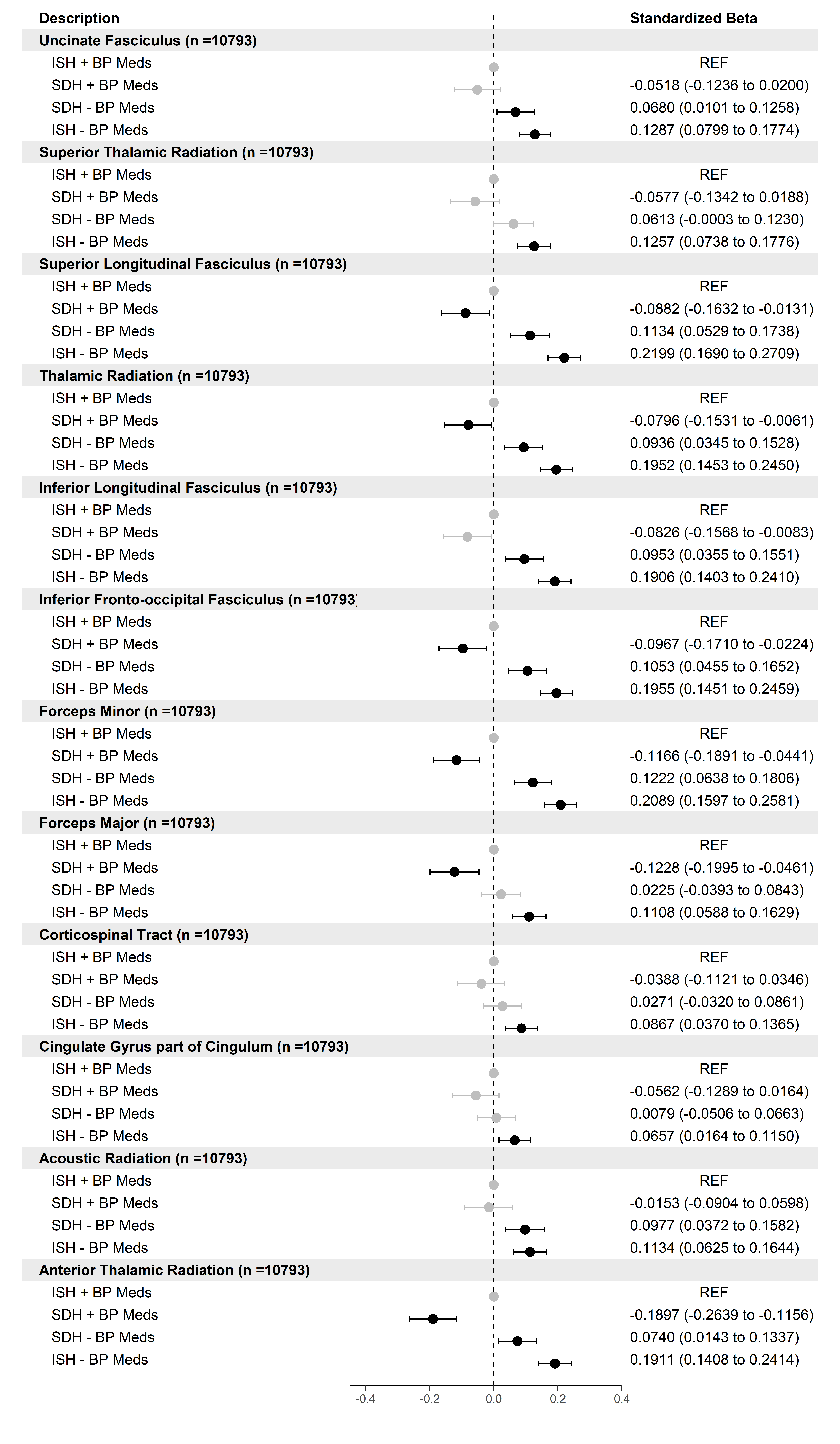


**Figure S11. Association between isolated systolic hypertension and systo-diastolic hypertension with brain microstructural white matter volumes for fractional anisotropy stratified by blood pressure medication use. Points in black are statistically significant (FDR p value < 0.05) standardized betas**


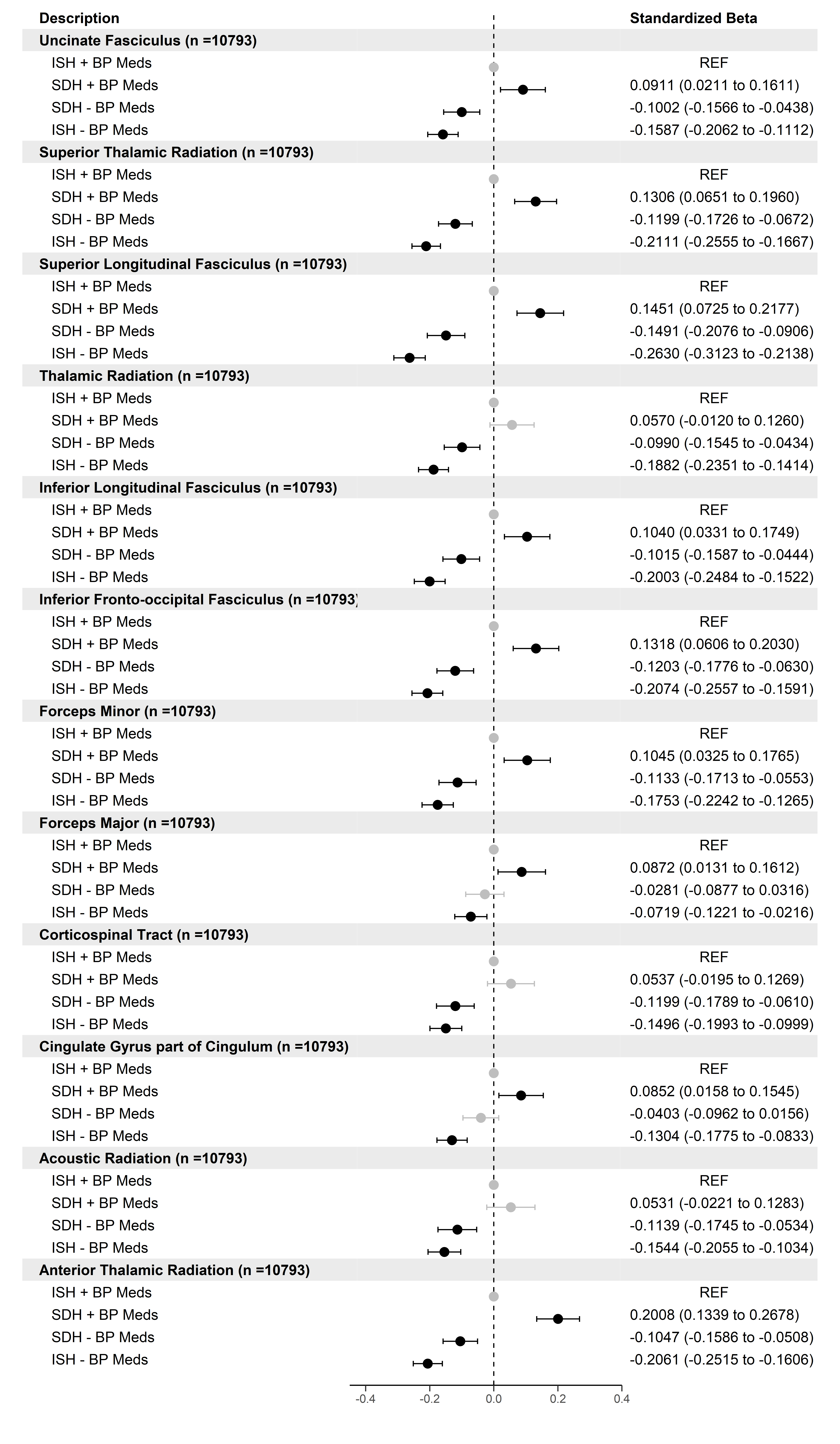


**Figure S12. Association between isolated systolic hypertension and systo-diastolic hypertension with brain microstructural white matter volumes for mean diffusivity stratified by blood pressure medication use. Points in black are statistically significant (FDR p value < 0.05) standardized betas**


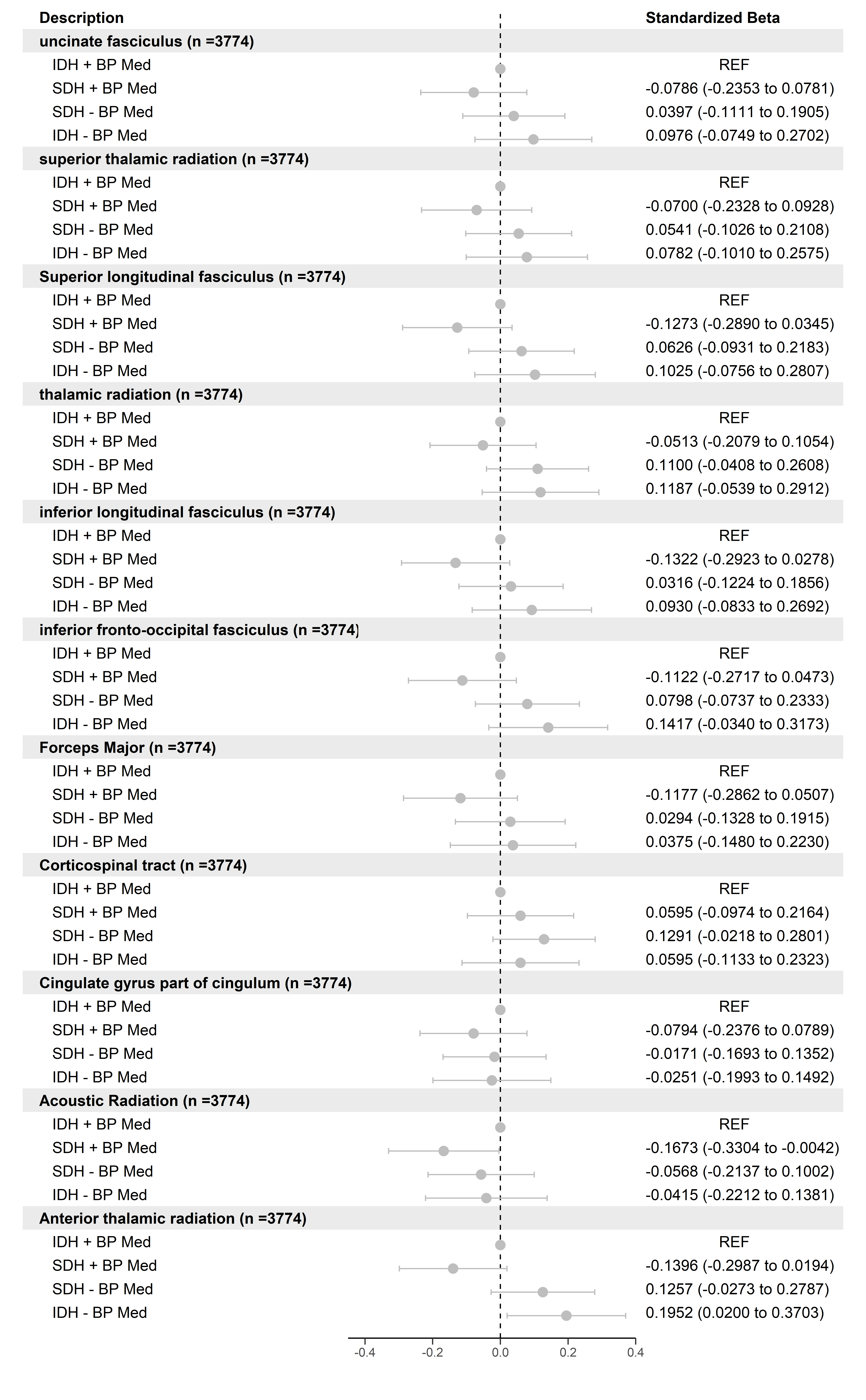


**Figure S13. Association between isolated diastolic hypertension and systo-diastolic hypertension with brain microstructural white matter volumes for fractional anisotropy stratified by blood pressure medication use. Points in black are statistically significant (FDR p value < 0.05) standardized betas**


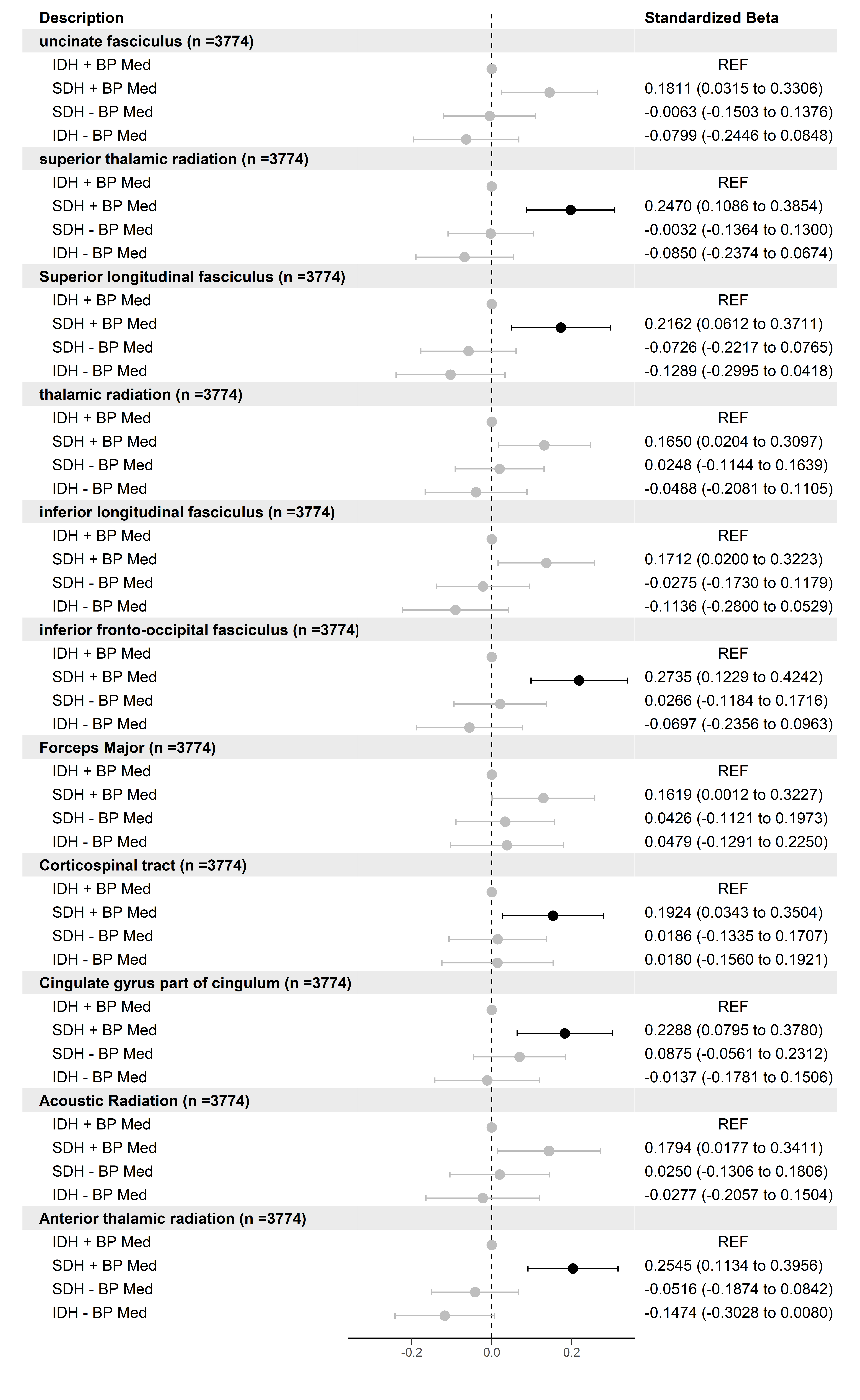


**Figure S14. Association between isolated diastolic hypertension and systo-diastolic hypertension with brain microstructural white matter volumes for mean diffusivity stratified by blood pressure medication use. Points in black are statistically significant (FDR p value < 0.05) standardized betas**
